# Neural growth patterns: how random and aligned fibers guide 3D cell organization and pseudospheroid formation

**DOI:** 10.3389/fbioe.2025.1659965

**Published:** 2025-10-03

**Authors:** Jana Hlinkova, Karolina Dziemidowicz, Mathilde M. Ullrich, Anne Eriksson Agger, Aina-Mari Lian, Janne Elin Reseland, Athina Samara

**Affiliations:** ^1^ Department of Biomaterials, FUTURE, Center for Functional Tissue Reconstruction, University of Oslo, Oslo, Norway; ^2^ Department of Tissue Engineering, Institute of Experimental Medicine, Czech Academy of Sciences, Prague, Czechia; ^3^ Department of Pharmaceutics, UCL School of Pharmacy, University College London, London, United Kingdom; ^4^ Department of Women’s and Children’s Health, Karolinska Institutet, Stockholm, Sweden; ^5^ Astrid Lindgren Children’s Hospital, Karolinska University Hospital, Stockholm, Sweden

**Keywords:** PCL, fibers, neuroblastoma cells, SH-SY5Y, aligned, random, SEM, pseudospheroids

## Abstract

**Background and purpose:**

Electrospun biomaterials replicate the structural complexity of the extracellular matrix (ECM), providing mechanical support and promoting neural cell survival and organization. Fiber orientation is a key determinant of neural cell behavior, influencing adhesion, migration, and differentiation. This study investigates how high seeding density combined with fiber directionality shapes SH-SY5Y culture morphology, gene expression, and early network formation; all critical factors for the design of next-generation scaffolds for neural tissue engineering.

**Methods:**

Polycaprolactone (PCL) scaffolds with either random or aligned fiber orientation were fabricated via monoaxial electrospinning. Human SH-SY5Y neuroblastoma cells were seeded at high density and cultured for 7 days, and cell viability was assessed by lactate dehydrogenase (LDH) activity. Neural, ECM, and differentiation markers were analyzed using quantitative PCR, Luminex cytokine profiling, and confocal immunofluorescence.

**Results:**

Hydrophobic PCL fibers supported cell adhesion, migration, and proliferation when cells were seeded in small clusters. After 7 days, cell coverage of the fiber-mat was significantly higher on random fibers compared to aligned ones (27.7% vs. 15.8%). Fiber orientation influenced both culture morphology and gene expression. Pseudospheroids formed on both substrates, that differed in perimeter (348.5 µm on random vs. 450.5 µm on aligned fibers, p < 0.05), with no significant difference in thickness (38.4 ± 7.7 µm vs. 43.2 ± 5.5 µm). mRNA expression of connexin 43 and β3-tubulin increased significantly from day 1 to day 7 on random fibers. On aligned fibers, mRNA patterns resembled cells cultured on glass (control), with elevated connexin 31 and doublecortin over time. Immunofluorescence showed early enrichment of nestin on aligned fibers (day 1), and greater expression of β3-tubulin, acetylated tubulin, and connexin 31 on aligned substrates, whereas fibronectin 1 was more prominent on random fibers.

**Conclusion:**

Fiber orientation significantly affected SH-SY5Y cell behaviour, including adhesion, formation of pseudospheroids, and differentiation marker expression under high-density conditions. Random and aligned fibers elicited distinct structural patterns and molecular responses, highlighting the importance of scaffold architecture in the rational design of neuroregenerative platforms. To our knowledge, this is the first study to describe scaffold-anchored neural pseudospheroids as a distinct model from conventional suspension spheroids.

## 1 Introduction

Fibrous scaffolds are central to biomedical engineering research, offering a 3D architecture that closely resembles native extracellular matrix (ECM), thereby creating a physiologically relevant environment for cell adhesion, growth, and networking (Carletti et al., 2011; Chan and Leong, 2008; Horwacik, 2022; Randhawa et al., 2024). Their structure not only guides the spatial arrangement of cells but also modulates temporal cellular responses (Bacakova et al., 2019) making them ideal for *in vitro* modeling of dynamic tissue regeneration. Owing to these properties, fibrous scaffolds have been widely applied in regenerative medicine as carriers of bioactive compounds for treatment (Chen et al., 2024; Braťka et al., 2022) to direct and understand vascularization, bone, cartilage, and ligament repair, and also for the localized delivery of therapeutics to the action site (Park et al., 2023; Zhang et al., 2022; Blahnová et al., 2020). In neural regeneration and cancer, fiber scaffolds offer directional cues for axonal guidance and neuronal growth, aiding tissue repair (Yang et al., 2005; McMurtrey, 2014; Tian et al., 2015; Kim et al., 2018; Kellaway et al., 2024).

Among the essential components of ECM, collagens are spatially organized into filaments that modulate tissue elasticity, and alignment affects tissue mechanical properties (Smith et al., 2018; Horwacik, 2022). The ECM also provides instructive spatial (morphological and topographical), and biochemical cues for cellular response and differentiation (Brown and Badylak, 2014; Smith et al., 2018), a function replicated by electrospun polymers, such as polycaprolactone (PCL) (Chew et al., 2008; Lee et al., 2005; Schnell et al., 2007; Yim et al., 2005; Gerardo-Nava et al., 2009; Marques-Almeida et al., 2024).

Unlike traditional 2D cell cultures, which constrain cells to a flat surface and limit physiological relevance, 3D cultures supported by scaffolds allow cells to grow in all dimensions, thus better mimicking the architecture, signaling, and microenvironment of native tissues. While culture media supplements traditionally support cell proliferation and differentiation, scaffold-based delivery of bioactive compounds via degradation or controlled release can further enhance these cellular processes by offering spatially and temporally regulated cues (Haidar and Eroglu, 2017; Sousa et al., 2020; Sousa et al., 2021; Duan et al., 2022; Katti et al., 2004; Melaiye et al., 2005). Moreover, scaffold topography, texture, and porosity may guide or drive cell fate, directly or indirectly modulating adhesion, growth, and function (Chan and Leong, 2008; Schaub et al., 2017; D’Amato et al., 2019; Zhang et al., 2022; Saghir et al., 2025). Fiber orientation also regulates gene expression (Ferraris et al., 2020; Werner et al., 2017), while porosity can promote nutrient and oxygen exchange, facilitate waste removal, and inhibit pathogen entry, making fibrous scaffold studies essential for neuroregenerative research (Khil et al., 2003; Fahimirad et al., 2021; Chen et al., 2022; Chamanehpour et al., 2024).

**FIGURE 1:**
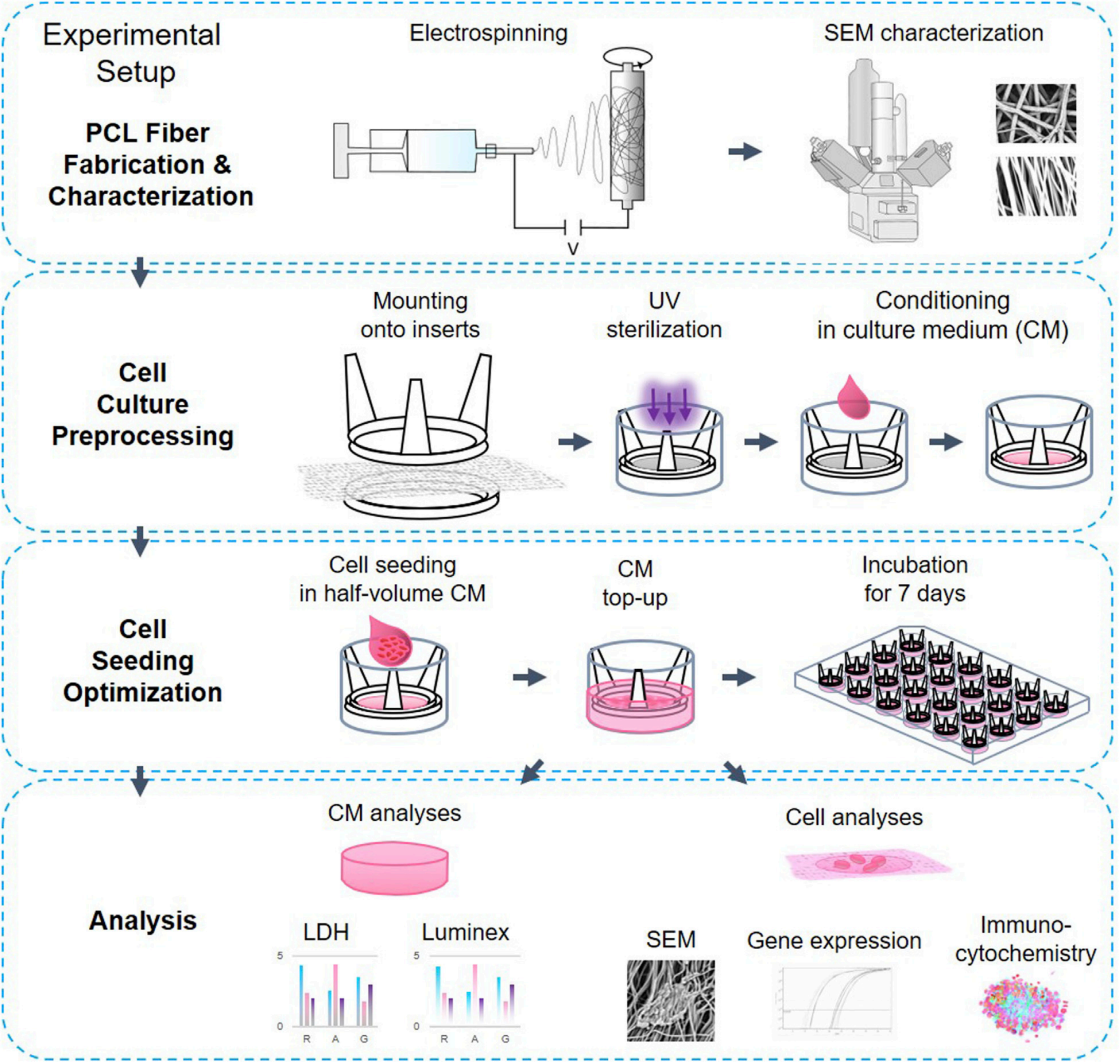
The experimental setup.

In this study, we employed inert electrospun PCL scaffolds with random or aligned fiber orientations, to assess their effect on neuronal-like SH-SY5Y cells seeded at high density as a proxy to tissue formation. PCL is a widely used scaffold material due to its stable degradation, pH neutrality, angiogenic properties, and minimal long-term inflammatory response (Edgar et al., 2016; Woodruff and Hutmacher, 2010; Dash and Konkimalla, 2012). Compared to smooth substrates like glass or plastic, organized PCL fibers offer a more ECM-relevant surface for cell attachment and growth (Gupta et al., 2009; Çapkin et al., 2012). The SH-SY5Y cell line is a well-characterized human neuroblastoma model used extensively to study proliferation, differentiation, and cell-material interactions on electrospun fibers (Senapati et al., 2024; Eleftheriadou et al., 2020; Jin et al., 2024; Hernández-Parra et al., 2024; Fiore et al., 2022). Previous studies largely focused on monolayer cultures (Huynh and Holsinger, 2023; Mungenast et al., 2023). We employed higher seeding densities to evaluate early 3D culture development as a proxy for tissue formation, to capture density-dependent cues that regulate network formation and signaling. This approach provides insight into how scaffold design can be optimized for neuroregenerative applications. Importantly, under these conditions we observed the formation of multicellular aggregates anchored to the scaffold surface, which we refer to as *pseudospheroids*. We introduce this term to distinguish scaffold-attached aggregates from conventional suspension spheroids, emphasizing their unique integration of material-derived and topographical cues in guiding tissue-like organization. The experimental set-up is illustrated on Figure 1.

## 2 Methods

### 2.1 Electrospun scaffold preparation

Electrospun fibers were fabricated using a NEU-BM (Tong Li Tech, China) electrospinning instrument with modifications from a previously published protocol (Dziemidowicz et al., 2023). In brief, PCL (∼80 kDa, Sigma-Aldrich, MA, United States) was dissolved at 12% w/v in hexafluoro-2-propanol (HFIP, Sigma-Aldrich, United Kingdom) and stirred until complete dissolution of the polymer overnight. The polymer solution was then loaded into 10 mL syringes and ejected through a 0.8 mm inner diameter blunt-end needle at 2 mL/h charged to 5–7 kV for 2.5 h and 2 h, to produce random and aligned fibers, respectively. The fibers were collected on baking paper on a −3 kV negatively charged mandrel (25 mm in diameter) spinning at 500 rpm and 3,750 rpm to reach random and aligned orientation, respectively at 14 cm distance from the spinneret to the collector. The spinneret was scanning parallel to the collector over a distance of 50 mm at 50 mm/s speed to uniformly distribute the fiber on the mandrel. All electrospinning experiments were conducted at room temperature (RT; 18 °C–23 °C) and at 35% ± 10% relative humidity.

### 2.2 Scaffold characterization

The fiber morphology was characterized using a TM3030 scanning electron microscope (Hitachi, Japan) at 15 kV and 100x/500x magnification, after sputter coating samples with gold for 60 s at a 20 mA current, on aluminum holders. When cells were seeded on the scaffolds, samples were fixed with 4% paraformaldehyde (PFA) for 15 min at RT, washed 3x for 5 min in phosphate saline buffer (PBS), rinsed in distilled water for 15 min, underwent alcohol dehydration grade (30%, 50%, 70%, 2% × 95%, 3% × 100%) for 5 min each and were left to dry overnight. The samples were sputter-coated with gold on carbon adhesive tabs and scanned the following day. Samples were analyzed in FIJI.

### 2.3 *In vitro* assessment

Neuroblastoma SH-SY5Y (ATCC) cells were routinely cultured in basal medium (Advanced DMEM:F12 (Gibco, MA, United States; 12634-010)) with 20% fetal bovine serum (FBS, Heat Inactivated, F9665, Sigma-Aldrich, MA, United States), 1% L-Glutamine and 1% Penicillin-Streptomycin (100 U/mL penicillin and 100 μg/mL streptomycin; 15140-122, Gibco, MA, United States) at 37 °C in 5% CO_2_. Cell seeding was optimized to obtain high monolayer confluency on glass slides by day 7 (D7). We selected day 1 (D1) and D7 as timepoints for the experimental days to investigate the difference in cell behavior to the surface material. D1 and D7 were chosen to capture early attachment and later network formation, respectively. The cells were counted using Countess 3 (Invitrogen by Thermo Fisher Scientific, United States) and 10^5^ cells were seeded onto glass cover slips (13 mm in diameter) or scaffolds fitted into CellCrown™ inserts (Scaffdex, Finland) for 24 well plate: The cells were seeded in small volume (ca 150 µL) reaching 1.7 × 10^5^/cm^2^ and left in incubator for 2 h to let the cells attach to the fibers. Later, the medium was topped up to 600 µL per well, and the cells were cultured for up to 7 days. Medium was changed on day 3. On experimental days 1 (D1) and 7 (D7) the supernatant medium was collected and stored at −20 °C for further assessment of LDH and cytokine release. The cell-laden scaffolds were visualised using SEM and quantification of mRNA expression change, and immunofluorescence of selected proteins via confocal microscopy.

### 2.4 Cytotoxicity assessment

Lactate dehydrogenase activity in the cell culture media was measured according to the manufacturer’s guidelines (11644793001, Roche, Switzerland). The working solution was prepared freshly before the testing and added 1:1 to the collected culture media thawed on ice, incubated on low rotation for 30 min on a shaker, protected from light for 30 min at RT. Absorbance was measured at 490 nm on a BioTek ELx800 absorbance microplate reader (BioTek, VT, United States). The LDH assay values of the cell medium of cell cultures on the fibers were normalized to the results of the LDH values of cells grown on glass.

### 2.5 Growth factor secretion assessment

The culture media were assessed for the content of fibroblasts growth factor (FGF-)1, FGF-2, follistatin and leptin using the Growth Factor Magnetic Bead Panel 1 (HAGP1MAG-12K 16-plex) (Merck, Kenilworth, NJ, United States). Factors were quantified with the Luminex 200 system (Luminex, TX, United States) employing xMAP technology and running Luminex xPONENT 3.1 software. The concentrations of the factors in the samples were calculated based on a logistic 5P weighted standard curve.

### 2.6 mRNA isolation and qPCR analysis

mRNA was extracted from cell lysate on ice using Dynabeads^®^ mRNA DIRECT™ Kit (cat. #61011, Ambion, Thermo Fisher Scientific, MA, United States) and transcribed to cDNA using cDNA kit ReverseAid First Strand cDNA Synthesis Kit (Ref K1622, Thermo Scientific, Lithuania) according to manufacturer’s guidelines using random hexaprimers. Differential mRNA abundance was quantified using 1 ng cDNA/reaction with 2x SybrGreen (SsoAdvanced Universal SYBR^®^ Green Supermix, cat.# 1725271, Bio-Rad, CA, United States) and a set of primers (Table 1) by qPCR (CFX Connect^®^ Real-Time System, Bio-Rad, CA, United States) run according to the manufacturer’s instructions. Ribosomal protein L19 *RPL19* was used as a housekeeping gene (Cai et al., 2007). We compared mRNA expression of several structural proteins and common neural and neuronal markers, namely actin (*ACTB*), β3-tubulin (*TUBB3*), nestin (*NES*) doublecortin (*DCX*), neural cell adhesion molecule (*NCAM*). We also assessed the mRNA expression of gap junctional proteins connexin 43 (Cx43, *GJA1*) and connexin 31 (Cx31, *GJB3*) and ECM proteins, namely filamentous collagen 1 and 3 (*COL1A1, COL3A1*) and glycoprotein fibronectin 1 (*FN1*), and adiponectin (*ADPN*) (Table 1). Biological triplicates in technical duplicates of each sample (glass, random and aligned fibers) from 3 separate experiments were each normalized to their respective surface results from D1. For comparison among the cells cultured on different surfaces, we normalized the data to *ACTB* (in Supplementary Data 1).

**TABLE 1:** List of primers used to detect and quantify abundance of mRNA of selected proteins.

Gene	Forward primer 5’ - 3′	Reverse primer 5’ - 3′
*hACTB*	TGC​TGA​TCG​TAT​GCA​GAA​GG	CCC​CCA​ATC​CAG​ACA​GAG​TA
*hADPN*	CTC​CTG​GGT​CCA​AGC​AAT​TA	CAT​GAC​GAA​ACC​CCA​TTT​CT
*hCOL1A1*	CAT​CTC​CCC​TTC​GTT​TTT​GA	CCA​AAT​CCG​ATG​TTT​CTG​CT
*hCOL3A1*	CTT​CTC​TCC​AGC​CGA​GCT​TC	TGT​GTT​TCG​TGC​AAC​CAT​CC
*hDCX*	TAT​GCG​CCG​AAG​CAA​GTC​TCC​A	CAT​CCA​AGG​ACA​GAG​GCA​GGT​A
*hFN1*	GAT​GCT​CCC​ACT​AAC​CTC​CA	CGG​TCA​GTC​GGT​ATC​CTG​TT
*hGJA1*	AAT​TCT​CGC​AGG​TAG​GCA​C	CCA​GAG​AGT​GTG​CAG​CAG​GT
*hGJB3*	CAA​TCA​CTT​GGC​GTG​ACT​TC	GTT​TGG​GCA​ACC​TTG​AGT​TC
*nNCAM*	GCG​ATT​GGT​GAA​CCT​GAA​AGT	TTG​CAC​TAC​TAC​CCT​CTG​TGT
*hNES*	TCT​GCG​AGC​CGC​TCC​CCT​TCT	GTG​CTT​CTC​CCC​GCC​AGC​GTT
*hRPL19*	GCG​GAA​GGG​TAC​AGC​CAA​T	CAG​GCT​GTG​ATA​CAT​GTG​GCG
*hTUBB3*	TCA​GCG​TCT​ACT​ACA​ACG​AGG​C	GCC​TGA​AGA​GAT​GTC​CAA​AGG​C

### 2.7 Immunofluorescence analysis by confocal microscopy

Cells were seeded on scaffolds and glass coverslips at a density of 100,000 (100k) and grown for 1 (D1) and 7 days (D7). At the experimental timepoints, the samples were fixed in 4% PFA for 15–45 min, followed by washing with PBS. Cells at day 7 were permeabilized with 0.1% Triton X-100 for 120 min, compared to 15 min for day 1 samples, to account for increased layer thickness and ensure adequate antibody penetration for confocal imaging. Samples were blocked with 1% bovine serum albumin (BSA) in PBS (BSA-PBS) for 1 h and incubated with the primary antibodies (diluted to working concentration in BSA-PBS as specified in (Table 2)) for 1 h at RT. Samples were washed 3x with PBS before incubating with fluorophore-conjugated secondary antibodies (goat anti-mouse Alexa 568 and Alexa 647, and donkey anti-rabbit Alexa568 (A11031, A21236, A10042, Invitrogen, Thermo Fisher Scientific, MA, United States) diluted 1:500 in BSA-PBS. After 1 h incubation, coverslips were 3x washed with PBS, incubated with FITC-conjugated phalloidin (Phalloidin-Alexa 488 1:200; A12379, Invitrogen, ThermoFisher, MA, United States) for 1h, 3x washed with PBS, incubated with 300 nM DAPI for 10 min, mounted with VectaShield (H-1000, Vector Laboratories, CA, United States) and sealed with clear nail polish. We analyzed filamentous actin (F-actin), acetylated tubulin, β3-tubulin, Cx31, DCX, 24-dehydrocholesterol reductase (DHCR24), FN1, nestin, and SRY-Box transcription factor (SOX2).

**TABLE 2:** List of primary antibodies used to visualize selected proteins.

Primary antibody	Dilution	Ref. No	Provider	Origin
Acetylated tubulin	1:1,000	sc-23950	Santa Cruz	Mouse
β3-tubulin	1:1,000	sc-80005	Santa Cruz	Mouse
Cx43	1:1,000	sc-271837	Santa Cruz	Mouse
Cx31	1:500	PAB3584	Abnova	Rabbit
DHCR24	1:500	PA5-27944	Invitrogen	Rabbit
Doublecortin	1:1,000	H00001641-M01	Abnova	Mouse
Fibronectin1	1:1,000	ab2413	Abcam	Rabbit
Fibronectin1	1:1,000	F3648	Sigma-Aldrich	Rabbit
Nestin	1:1,000	ab105389	Abcam	Rabbit
SOX2	1:500	sc-365823	Santa Cruz	Mouse

Immunofluorescent samples were viewed by confocal microscopy using a Leica SP8 upright microscope fitted with HyD and PMT detectors, using objective 40x HC PL Apo CS2 40x/1.3 (Leica Microsystems, Germany). Excitation laser lines at 405, 488, 552, and 650 nm were used for DAPI, Alexa488, Alexa568, and Alexa 647 respectively in Leica Application Suite X, (version 3.5.6.21594, Leica Microsystems, Germany).

### 2.8 Image analyses

Collected images were analyzed using FIJI (Schindelin et al., 2012), and its plug-ins Bio-Format (Linkert et al., 2010) and 3D ImageJ Suite (Schmid et al., 2010). The confocal images were prepared for publication in Z and 3D projections.

SEM micrographs (n = 6 per fiber type and timepoint) were used to evaluate cluster size, morphology, migration, and coverage of the fibrous mat by the cells by measuring cell-covered areas on the fibers.

To evaluate cell cluster thickness and its incorporation to the fiber mat on D7, Z dimensions of 10 randomly selected 3D cell clusters that fitted scanning area per experimental group were marked and analyzed in micrographs from confocal microscopy via the Leica Application Suite X.

Mitotic index for D1 was calculated as a ratio of mitosis, manually counted, to total number of cells from 5 representative 3D micrographs from confocal microscopy in 3D ImageJ Suite, FIJI. Mitotic index for D7 was presented as total mitosis per scanned area due to technical limitations of the automated cell count. For simplification, D1 mitotic index was recalculated per scanned area.

### 2.9 Statistical analyses

Statistically significant differences in the quantifiable parameters were estimated using analysis of the variance (ANOVA, GraphPad Prism 9, CA, United States) with a confidence interval of 95% for group comparison in case of normal Gaussian distribution with Tukey post-hoc test, and as min–max and with z25 and z75% and nonparametric Kruskal-Wallis post-hoc test otherwise. In the case of comparison only of 2 groups, we used either a parametric t-test or nonparametric Mann-Whitney t-test, comparing ranks, depending on data distribution. Values of p ≤ 0.05, p ≤ 0.01, and p ≤ 0.001 were considered statistically significant among the groups that were compared. The data are presented as the mean ± standard deviation (SD) and the averaged values were determined from 3 independently prepared biological samples.

## 3 Results

### 3.1 Random and aligned PCL fiber fabrication and characterization

**FIGURE 2:**
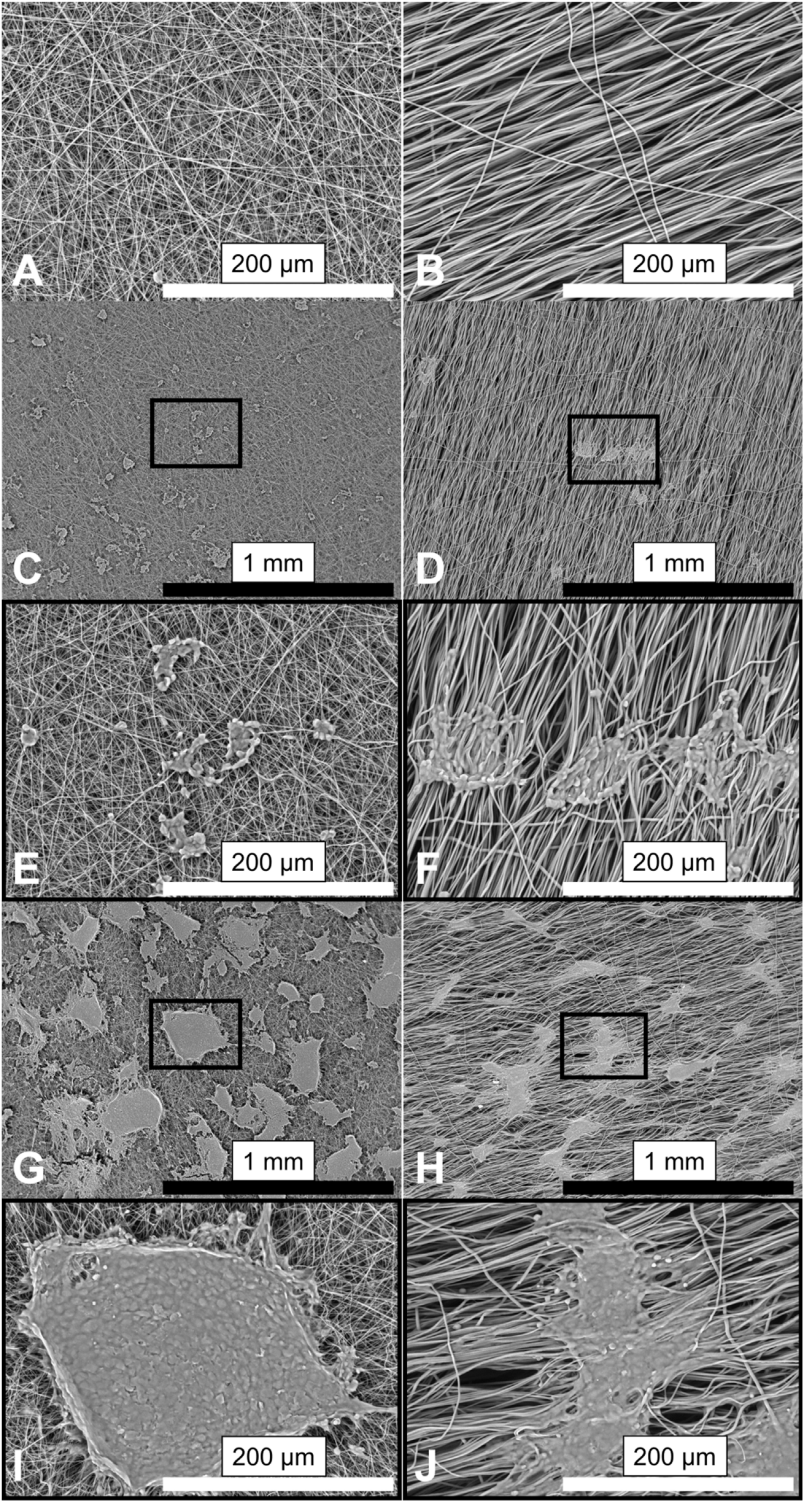
SEM micrographs showing the morphology of PCL fibers and morphology of cells seeded on them. Representative images (n = 6) of the PCL scaffolds: randomly oriented fibers **(A)**; (left) and aligned fibers **(B)**; (right). SH-SY5Y cells cultured on fibers for 1 day **(C–F)** and after 7 days in culture **(G–J)**. Insets **(E,F)** for **(C,D)**; **(I,J)** for **(G,H)** correspond to magnified views of the marked areas. Images are shown at 100× (top) and 500× (bottom) magnification. Scale bars: black = 1 mm; white = 200 µm.

**FIGURE 3:**
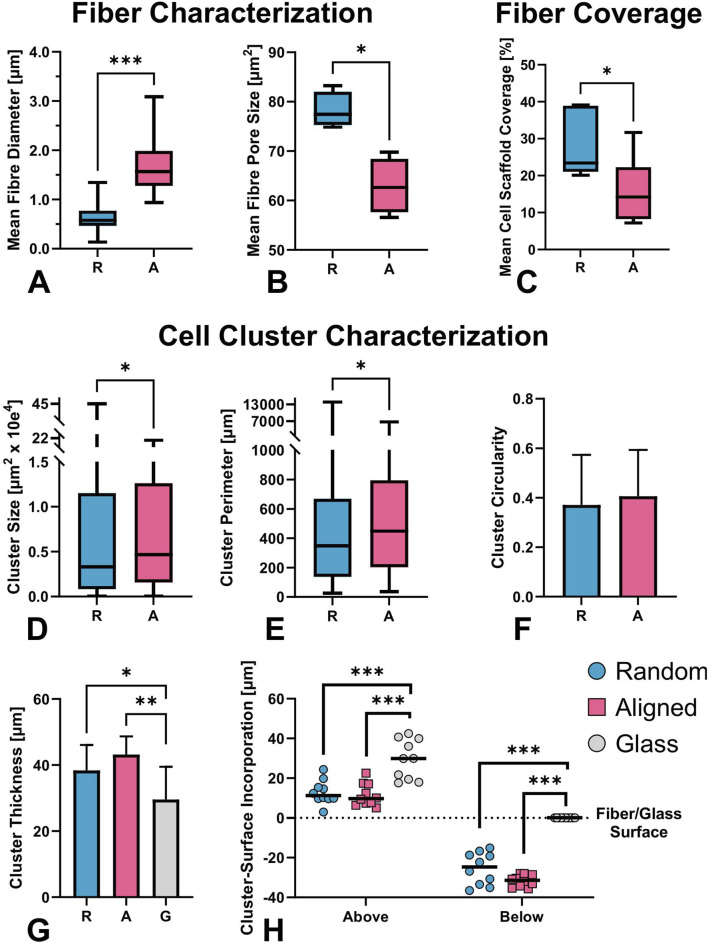
SEM-based characterization of electrospun fiber mats and quantification of cell–scaffold interactions on day 7 (D7); **(A-C)** n = 6). **(A)** Average fiber diameter and **(B)** pore size of aligned and random fibers. Aligned fibers displayed a slightly wavy morphology with larger diameters compared to the straight randomly organized fibers (mean 1.65 ± 0.48 µm, vs. 0.63 ± 0.24 µm, respectively, p < 0.001). The former offered significantly smaller pore size (median 62.64 vs. 77.4 µm^2^, p < 0.029), as the pores of the aligned fibers follow the underlying alignment structure, with less crossover and interweaving. **(C–H)** Quantitative assessment of cell-material interaction from 3D confocal micrographs (D-H, n = 10): percentage of scaffold surface covered by cells **(C)**, cluster size **(D)**, cluster perimeter **(E)**, cluster thickness **(F)**, circularity **(G)**, and spatial distribution of clusters above and below the fiber plane **(H)**. Despite the smaller pore size in aligned mats, cells covered a significantly smaller percentage of the surface compared to random fibers. Differences in fiber orientation and diameter led to significantly distinct cluster morphology and distribution. Significant differences marked as *p ≤ 0.05, **p ≤ 0.01, ***p ≤ 0.001.

Aligned and random orientation morphologies (Figures 2A,B) were characterized by SEM from (Figures 3A,B, Fiber Characterization panel).

#### 3.1.1 Fibers support attachment and proliferation of neuroblastoma cells

Cells adhered to fibers from day 1 (D1) to day 7 (D7), forming mat-like pseudospheroids that were morphologically similar across substrates but more frequent and expansive on random fibers. Aligned fibers supported deeper scaffold integration, and further analysis revealed distinct clustering dynamics compared to uniform spreading on glass. Notably, aligned fibers restricted cell–cell connectivity, due to strong directional cues, deviating from the shortest-distance connection typically observed on glass and random fibers. An increase in cluster size and fiber coverage was documented on both fiber types over the 7-day period (Figures 2C–J). On aligned fibers, cell clusters seemed to physically pull individual fibers as early as D1, altering their orientation (as clearly visible in SEM images, on Figures 2D,F,H). FIJI analysis revealed non-significantly lower fiber coverage on aligned fibers at D1, but significantly lower coverage by D7 (Figure 3C, Fiber Coverage panel).

**FIGURE 4:**
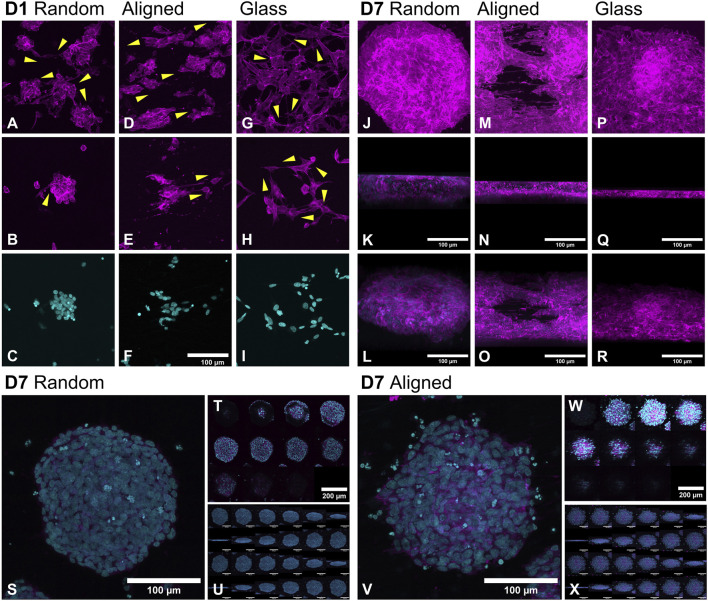
Morphological changes in SH-SY5Y cell organization on random, aligned fibers, and glass at day 1 (D1); **(A–I)** and day 7 (D7; **(J–X)** visualized by confocal microscopy. On D1, cells seeded on glass **(G–I)** formed an even monolayer, while both random **(A–C)** and aligned **(D–F)** PCL fibers promoted early clustering. By D7, high-density cultures developed into pseudospheroids and thick multilayer mats on both fiber types **(J–O)**. Cells on random fibers and glass exhibited interconnectivity consistent with shortest-distance principles, while on aligned fibers, interactions followed the fiber axis (indicated by yellow arrows). Pseudospheroid morphology varied by topography: random fibers supported more circular structures with flatter bases **(S–U)**, whereas aligned fibers yielded deeper, cone-shaped aggregates with less defined outlines **(V–X)**. F-actin is stained with fluorescent phalloidin (magenta); nuclei are stained with DAPI (cyan). Scale bars: 100 µm unless indicated (T, W = 200 µm).

On D7, the number and size of cell clusters differed significantly (Figure 3, Cell Cluster Characterization panel). We counted 331 clusters on random fibers with a median area of 0.3 µm^2^, and 170 clusters on aligned fibers with a median area of 0.5 µm^2^, over an analyzed surface area of 2.25 mm^2^ (non-Gaussian distribution, p = 0.020; Figure 3D). Cluster perimeter was also significantly larger on aligned fibers (p = 0.013; Figure 3E), with medians of 348.5 µm (random) and 448.4 µm (aligned). These, not fully globular, pseudospheroid structures eventually merged into thick, multilayered mats on both substrates. (Figure 4M). Circularity analysis from representative images (n = 43 for random, n = 33 for aligned) showed normally distributed values with no significant differences (0.37 ± 0.20 vs. 0.41 ± 0.19, respectively; Figure 3F).

### 3.2 Confocal microscopy indicates topographic influence on cytoskeletal architecture and nuclear distribution

Confocal microscopy following FITC-phalloidin and DAPI staining enabled analysis of cytoskeletal architecture and nuclear distribution within clusters across the two fiber types. No significant difference in overall cluster thickness was observed between fiber types (38.4 ± 7.7 µm on random vs. 43.2 ± 5.5 µm on aligned, n = 10), while glass supported significantly lower vertical stratification (29.6 ± 9.9 µm; p = 0.0486 and p = 0.002 vs. random and aligned, respectively). Clusters on aligned fibers penetrated deeper into the mat (31.6 ± 3 µm vs. 25.5 ± 8 µm random; p = 0.04), though the protruding part above the fiber mat was similar (12.9 ± 6 µm vs. 11.5 ± 5.8 µm). The relative incorporation depth (as a percentage of total cluster thickness) was higher on aligned fibers (74.1% ± 10%) compared to random (66.16% ± 15%), but this difference was not statistically significant (Figure 3H).

Figures 4A–I shows cell morphology on D1, while Figures 4J–X depicts D7 outcomes. On D1, cells formed uniform monolayers on glass (Figures 4G–I) but clustered on fibers (Figures 4A–F). Over time, these aggregates expanded into larger structures (Figures 4J–O). On D7, glass-supported cultures retained a largely monolayer architecture with some vertical stacking (Figures 4P–R). In contrast, fiber-based cultures formed compact pseudospheroids (Figures 4J–L) and thick multilayer formations (Figures 4M–O). On random fibers, pseudospheroids were more defined and circular (Figures 4S–U), with a preserved dome-like structure at the base. On aligned fibers, clusters appeared more dispersed, often integrating deeper into the scaffold and decreasing in volume (Figures 4V–X). Pseudospheroids commonly reached diameters of ∼250 µm but remained under 50 µm thick, consistent with spheroidal proportions along the Z-axis. On glass, SH-SY5Y cells exhibited typical hexagonal morphology with broad, flat protrusions on D1 (Figure 4H), whereas cells on fibers displayed elongated shapes conforming to the underlying topography (Figures 4A,D). On random fibers, protrusions were primarily connected to nearby clusters along the shortest path (Figure 4B), similar to those observed on glass (Figures 4G,H). However, on aligned fibers, inter-cluster communication was limited; protrusions predominantly extended along the fiber axis, contacting clusters on the same fiber rather than following minimal-distance paths (Figures 4D,E,M). Prominent lamellipodia, reminiscent of neurites, aligned tightly to fiber directionality by D7 (Figure 4M).

Nuclear morphology also varied with substrate. On glass, nuclei were slightly elongated and oriented toward neighboring cells. On fibers, cells appeared rounder with nuclei conforming to scaffold topography; globular on random fibers (Figure 4C) and elongated on aligned ones (Figure 4F). Increased apoptotic nuclei were noted along the periphery of clusters formed on aligned fibers (Figure 4V). Interestingly, in D7 pseudospheroids, nuclear orientation remained random unless in direct fiber contact, indicating a more localized topographic influence.

### 3.3 Cell proliferation rate is not affected by surface morphologies

**FIGURE 5:**
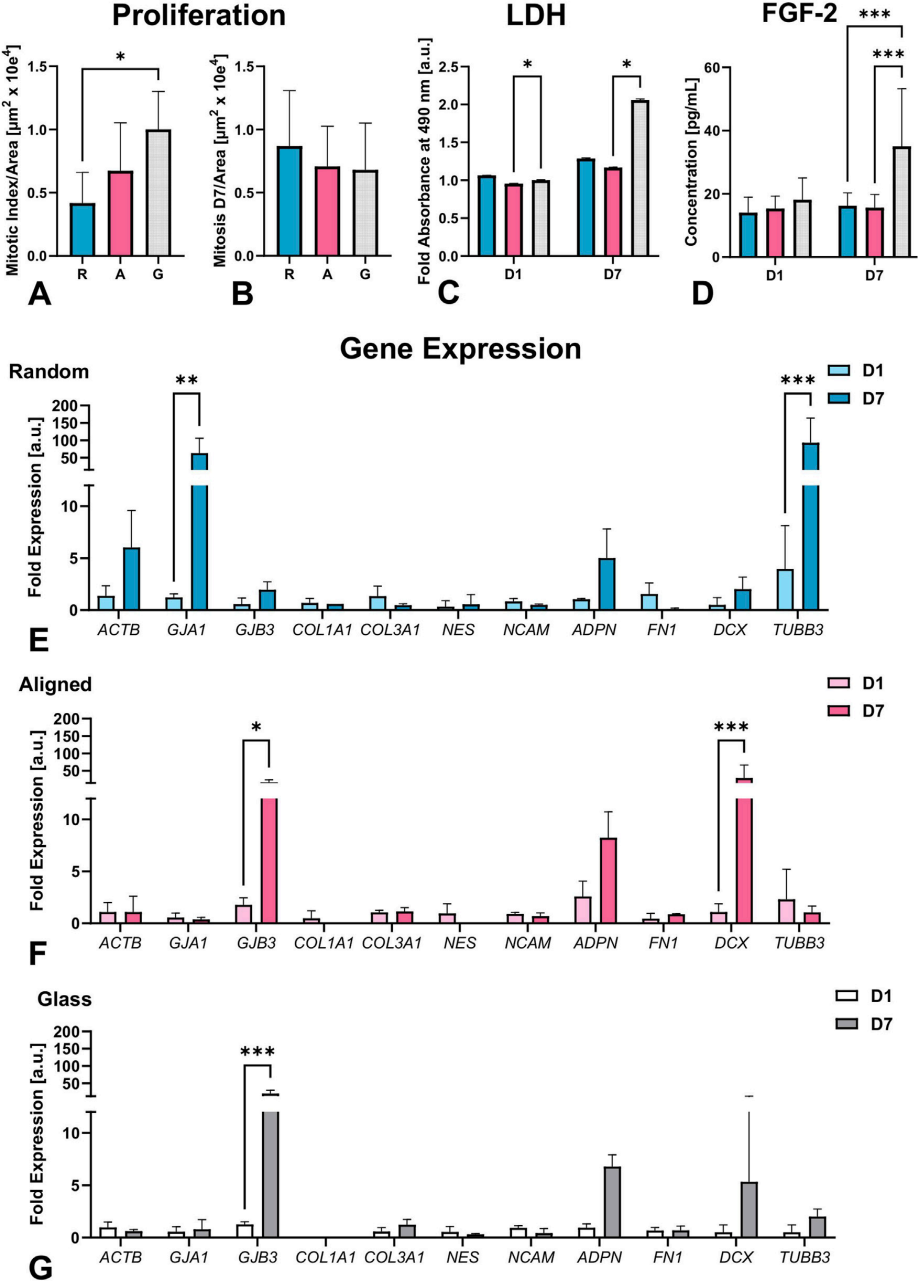
Quantitative assessments include mitotic index per area on D1 **(A)** and D7 **(B)**, LDH activity **(C)** on D1 and D7 normalized to glass-D1 controls, and FGF-2 concentration on D1 and D7 **(D)**. Cells proliferated significantly less on random fibers compared to glass. LDH assay indicated active metabolism and confirmed material non-cytotoxicity, with the highest LDH values on glass. FGF-2 levels remained stable on fibers but doubled on glass by D7, suggesting a proliferation-related response, n = 3 sets of experiments, total 10 biological replicates. qPCR analysis of gene expression normalized to RPL19. Cells grown on random fibers **(E)** showed significantly increased expression of actin (*ACTB*), β3-tubulin (*TUBB3)*, and connexin 43 (*GJA1*). In contrast, cells on aligned fibers **(F)** and glass **(G)** exhibited elevated expression of doublecortin (*DCX)* and connexin 31 (*GJB3*). Nine biological replicates were analyzed from 3 separate experiments. Significant differences are indicated as follows: *p ≤ 0.05, **p ≤ 0.01, ***p ≤ 0.001.

To quantify the surface effect on cell proliferation, we assessed the mitotic index using confocal micrographs (n = 5, Figures 5A,B, Proliferation panel), lactate dehydrogenase (LDH) activity to evaluate cytotoxicity (Figure 5C, LDH panel), and cytokine release assessment (Figure 5D, FGF-2 panel). The mitotic index was significantly lower on random fibers compared to the glass surface. On average, the total number of cells on D1 was comparable between groups grown on fibers (149.4 ± 9.7 random vs. 145.4 ± 28.0 aligned, p > 0.99) with fewer cells on glass (127.2 ± 31.2. p = 0.31 vs. random, p = 0.77 vs. aligned).

Due to the dense organization of pseudospheroids on D7, automatic segmentation of total cell counts using FIJI’s 3D Image Suite was not feasible. Therefore, mitotic figures were quantified per scanned area and compared across conditions. The mitotic index on D1 (expressed as mitoses per 10^6^ μm^2^) was significantly lower on random fibers (0.42 ± 0.24) than on glass (1.00 ± 0.30; p = 0.03), with intermediate values on aligned fibers (0.67 ± 0.38; Figure 5A). No significant differences were found across substrates on D7 (random: 0.87 ± 0.44; aligned: 0.71 ± 0.32; glass: 0.68 ± 0.37; Figure 5B), although the trend was reversed.

The LDH assay, performed across three independent experiments with a total of 10 biological replicates, showed slightly lower values for cells on aligned fibers compared with random fibers at both D1 and D7; however, these differences were not statistically significant (Figure 5C, LDH panel), yet the values of aligned fibers were statistically significantly reduced when compared to glass on both, D1 and D7 (p = 0.04 and p = 0.04, respectively). The cells seeded on glass reached the highest cell confluency among the testing groups as visualized by confocal imaging, suggesting that LDH results reflected the metabolic activity of the alive cells rather than cell death caused by the cytotoxicity of the material.

LDH assay results showed slightly lower values on aligned fibers compared to random fibers on both D1 and D7, but these differences were not statistically significant (Figure 5C, LDH panel). In contrast, LDH release on aligned fibers was significantly reduced compared to glass at both timepoints (p = 0.04; Figure 5C), indicating decreased cell membrane damage on aligned scaffolds. Confocal imaging confirmed that cells on glass achieved the highest confluency. Thus, the elevated LDH levels on glass may reflect increased cell density, stress from crowding, or higher turnover, rather than direct material-induced cytotoxicity. Luminex cytokine analysis from 3 separate experiments containing 10 biological replicates total revealed stable release of FGF-2 on both fiber types, while a significant increase was observed on glass by Day 7 (p < 0.001; Figure 5D, FGF-2 panel), likely reflecting higher confluency due to limited surface area. SH-SY5Y cells under these conditions did not engage in acute wound-like signaling pathways, as indicated by the absence of EGF, angiopoietin-2, G-CSF, BMP-9, endoglin, endothelin-1, leptin, follistatin, VEGF-C, and VEGF-D.

### 3.4 Fiber organization affects cell differentiation

We observed topography-dependent differences in mRNA expression directed by fiber organization, as shown in the Gene Expression panel (Figures 5E–G). To assess how 3D formation affects ECM remodeling and cell communication, we analyzed the expression of structural, neural, gap junctional, and ECM-related genes, along with stress marker adiponectin (*ADPN*) (Jung et al., 2006) in 3 sets of separate experiments in biological triplicates and technical duplicates.

On random fibers (Figure 5E), mRNA expression of actin (*ACTB*) and β3-tubulin (*TUBB3*) significantly increased from D1 to D7, up to 6-fold and over 20-fold, respectively (p < 0.001). In contrast, mRNA abundance of these markers remained relatively stable on aligned fibers, and glass (Figures 5F,G). Nestin (*NES*) expression showed little change in random fibers, slight downregulation on glass, and a notable decrease on aligned fibers by D7 (Supplementary Data 1C). *NCAM* levels were similarly stable across all conditions.

*DCX* mRNA expression increased on all substrates, with a modest 3-fold increase on random, 5-fold on glass, and a 30-fold increase on aligned fibers (p < 0.001). However, when normalized to *ACTB,* DCX expression decreased over time on random and glass, and remained stable on aligned fibers (Supplementary Data 1D).

Gap junction markers showed inverse patterns. Connexin 43 (*GJA1*) increased significantly, up to 60-fold on random fibers by D7 (p < 0.01) but remained unchanged on aligned fibers and glass. In contrast, connexin 31 (*GJB3*) increased markedly on glass (∼20-fold, p < 0.001) and aligned (∼15-fold, p < 0.05), with only a modest rise on random (∼2-fold) (also supported by Supplementary Data 1A).

*Fibronectin (FN1)* mRNA expression decreased on random fibers but stayed stable on aligned and glass surfaces (Supplementary Data 1C). Collagen mRNA expression varied: *COL1A1* was undetectable on glass and minimally expressed on fibers, while *COL3A1* increased on glass, decreased on random, and was stable on aligned (Supplementary Data 1A).

*ADPN* mRNA expression increased across all substrates, with the greatest fold change on glass (∼7-fold), followed by random (∼5-fold) and aligned (∼3-fold) fibers Supplementary Data 1B. GAPDH expression also rose from D1 to D7, with high variability, peaking at ∼350-fold on random, >100-fold on glass, and ∼20-fold on aligned fibers (not shown).

In summary, mRNA analysis revealed substrate-driven differences in gene expression, particularly among cytoskeletal (*ACTB, TUBB3*), gap junctional (*GJA1, GJB3*), and early differentiation and migration marker *DCX*. These patterns reflect how scaffold architecture can distinctly influence early neural gene programs and intercellular communication.

### 3.5 Fiber organization alters protein expression in pseudospheroids

**FIGURE 6:**
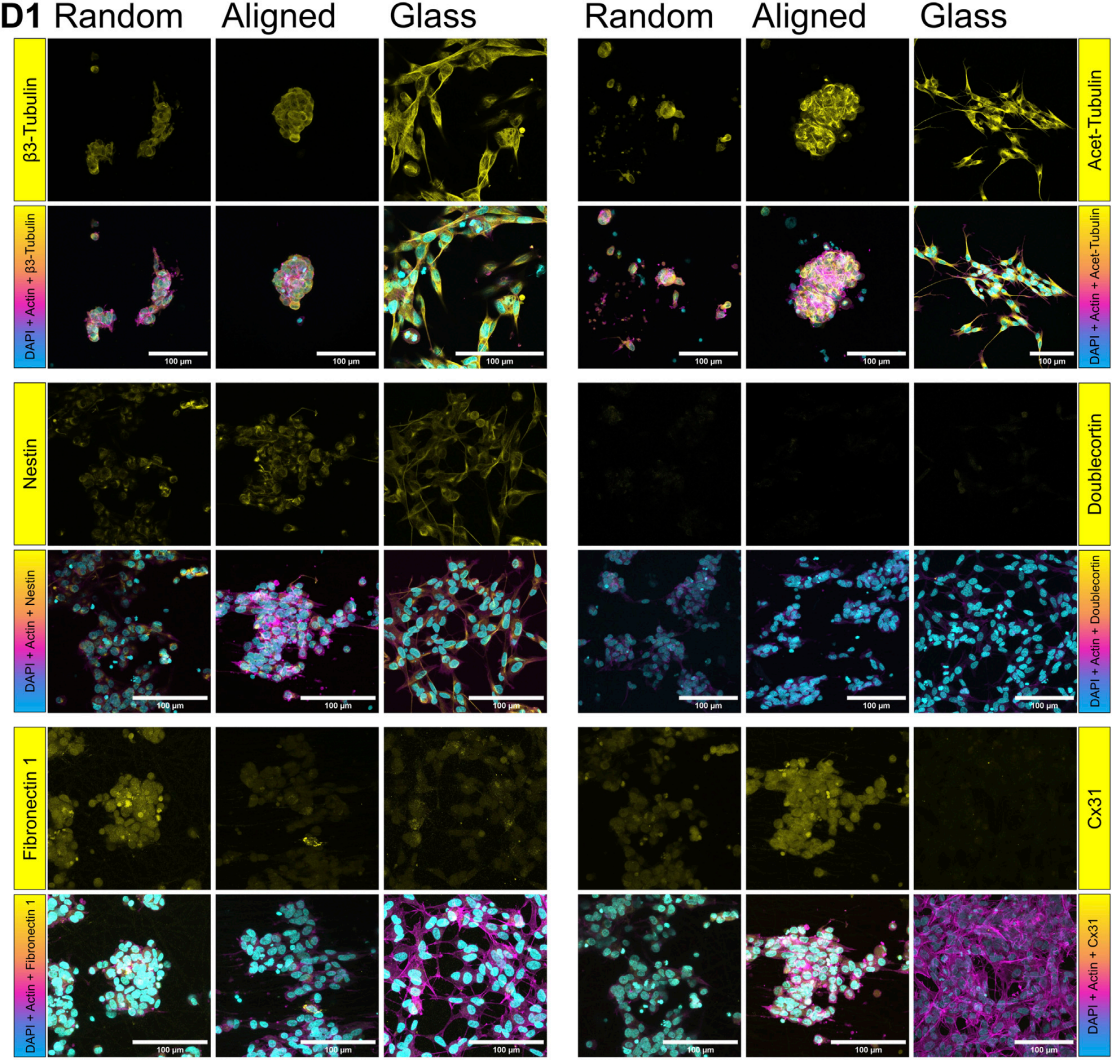
Protein expression on SH-SY5Y cells grown on random and aligned fibers, and glass on D1. Yellow shows β3-tubulin, acetylated tubulin, nestin, doublecortin (DCX), fibronectin 1, and connexin 31 (Cx31). F-actin (magenta) is stained by fluorescent phalloidin, nuclei (cyan) by DAPI.

**FIGURE 7:**
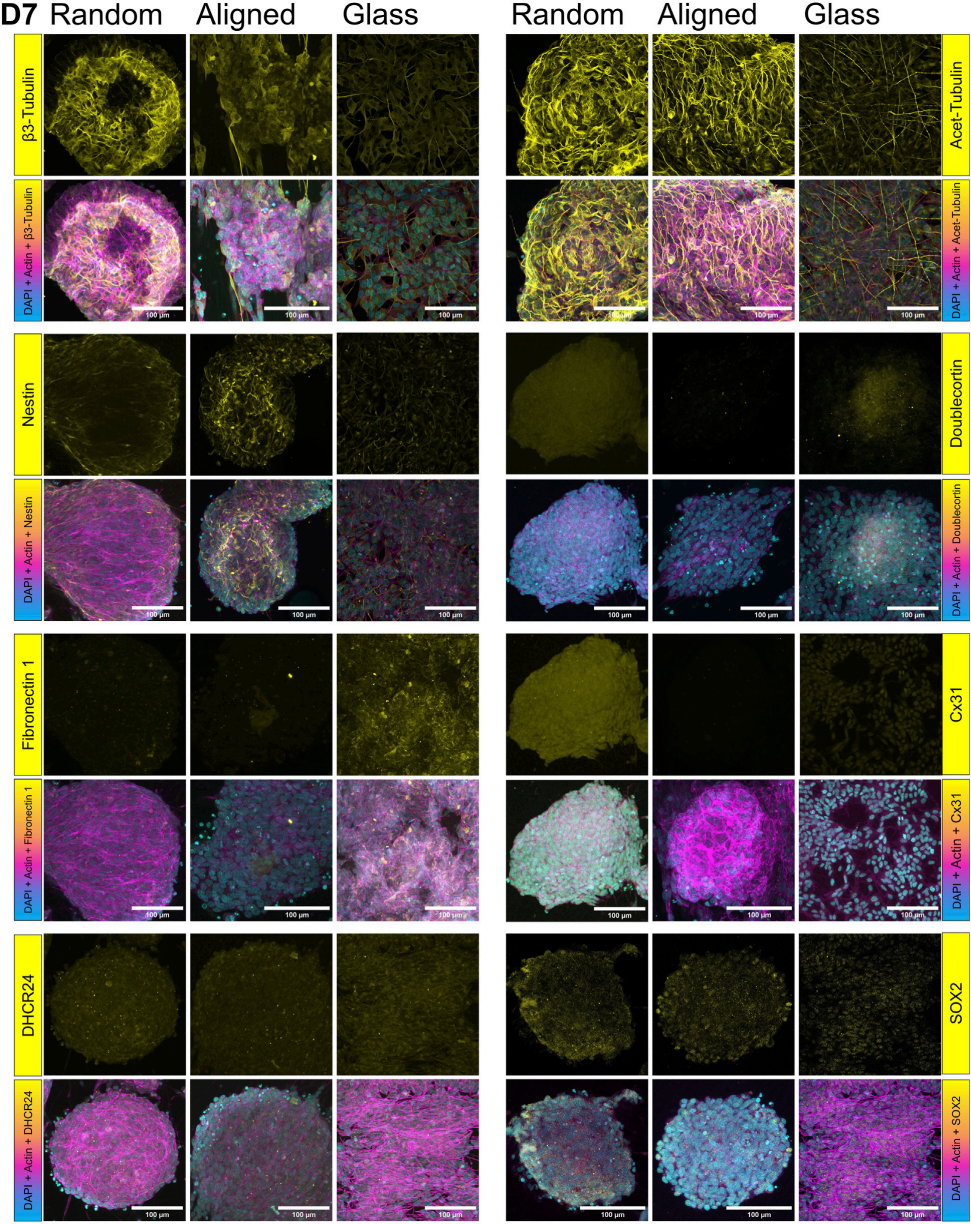
Protein expression in SH-SY5Y cells cultured on random and aligned fibers, and glass, at day 7 (D7): β3-tubulin expression increased on random fibers, while nestin was elevated on aligned fibers. FN1 expression decreased on random fibers at D7, whereas Cx31 signal increased. However, DCX and Cx31 were not detected by immunohistochemistry (IHC) on aligned fibers at D7. Target proteins (β3-tubulin, acetylated tubulin, nestin, doublecortin (DCX), fibronectin 1 (FN1), connexin 31 (Cx31), DHCR24, and SOX2) are shown in yellow. F-actin is visualized in magenta via fluorescent phalloidin staining.

To assess cell and pseudospheroid morphology across the different substrates, we first performed 3D cytoskeletal and nuclear imaging using fluorescent phalloidin and DAPI staining, followed by confocal microscopy (D1 in Figure 6, D7 in Figure 7).

Aiming to examine protein localization within the pseudospheroids, we carried out immunofluorescent staining for two well-established markers of neuronal identity and cytoskeletal remodeling, namely acetylated and β3-tubulin (top panels, Figures 6, 7). Acetylated tubulin marks stabilized microtubules critical for cytoskeletal integrity and mitotic spindle function, especially in post-mitotic and elongating cells (Nekooki-Machida and Hagiwara, 2020) and β3-tubulin is a microtubule element associated with early neuronal differentiation (Spildrejorde et al., 2023). Acetylated tubulin was abundantly expressed on both fibrous substrates but showed lower expression on glass. On D7, cells on random fibers exhibited more uniform and widespread acetylated tubulin staining, whereas cells on aligned fibers displayed a distinct network of thin cytoplasmic extensions. The expression pattern of β3-tubulin on D7 was consistent with the qPCR findings. DCX expression increased from D1 (Figure 6) to D7 (Figure 7) on both glass and random fibers in agreement with qPCR data, while no such increase was observed on aligned fibers (see Supplementary Data 1A). Nestin expression remained stable from D1 to D7 on both fiber types but declined on glass by D7, also matching qPCR trends (middle panel on Figure 5, second panel on Figure 7). FN1 protein expression decreased on fibers between D1 (Figure 6) and D7 (Figure 7), while remaining stable on glass, mirroring mRNA results (lower panel on Figure 6, third panel on Figure 7).

On D7, we also detected DHCR24 and SOX2 expression (Figure 7). DHCR24 (24-dehydrocholesterol reductase) is involved in cholesterol biosynthesis and implicated in neuroprotection and cellular stress response. SOX2 is a transcription factor essential for maintaining neural progenitor identity and pluripotency, and its presence may indicate retention of undifferentiated or stem-like characteristics within the culture (Samara et al., 2022). DHCR24 was evenly expressed in cells on glass and random fibers but was restricted to the periphery of pseudospheroids on aligned fibers (Samara et al., 2014). SOX2 was observed in the nuclei of cells on aligned fibers and glass but was predominantly cytoplasmic in cells on random fibers, particularly at the edges of pseudospheroids.

Overall, the confocal microscopy data support the SEM observations and gene expression results, confirming distinct substrate-specific effects on cytoskeletal organization, stress responses, and early differentiation markers.

## 4 Discussion

Employing a comprehensive experimental configuration, we investigated the impact of the directionality of PCL fibers on cell–fiber interactions and the organization of SH-SY5Y neuronal-like cells. We seeded cells at high densities on aligned and randomly arranged fibers, where the latter mimic the extracellular matrix (ECM) more closely, and characterized adhesion, differentiation, and morphology over a 7-day period. Cells clustered into 3D pseudospheroids with thickness reaching approximately one-fifth of their diameter. Notably, random fibers promoted significantly larger surface coverage, while aligned fibers yielded larger and more elongated clusters.

The findings of this work both build on and extend insight on topography cues leading to neural network formation of prior studies. Our study suggests that random fibers, due to their larger pore size and thinner diameters, facilitated greater surface coverage. Electrospun PCL fiber scaffolds have previously shown that variations in fiber diameter and pore size affect cell behaviour (Ibrahim and Klingner, 2020; Schiffman and Schauer, 2008), with fiber diameter documented to influence rigidity and elasticity (Doss et al., 2020). Aligned fibers, despite being wavy and not completely linear, offered a more elastic interactive surface, that enhanced structural elongation and cluster depth compared to the random fibers and glass, where cells could deform and stretch, as shown on the SEM images on D7, and previously observed (Hall et al., 2016).

Despite the differences in diameter and pore size, confocal imaging on D1 showed no significant difference in initial cell attachment across substrates, suggesting fiber architecture, rather than mechanical constraints alone, influenced subsequent cell behaviour. Previous work similarly observed no significant changes in relative proliferation of the cells between 0.75 and 1.45 μm fiber diameter (Christopherson et al., 2009). They also documented increased β3-tubulin protein expression on the thicker fibers, and 3D-like cell structure formation, as we did. The mean diameter of both the samples used in our study (0.63 vs. 1.65 μm, random vs. aligned, respectively) supports similar cell adhesion and proliferation.

The formation of pseudospheroids aligns with other 3D culture models, such as gold nanostructure-induced spheroids reaching ∼300 µm in diameter by D7 (Suhito et al., 2021). SEM analysis at D7 revealed low circularity values (0.37 for random, 0.4 for aligned), suggesting cluster fusion into broad, multilayer mats. These mat-like structures exhibited cell protrusions and lamellipodia that already connected clusters on day 1 (D1). Cells on random fibers and glass followed the principle of shortest-distance connectivity, whereas on aligned fibers, cell interactions were guided by fiber directionality. (Hammarback and Letourneau, 1986; Papadopoulou et al., 2010).

Interestingly, limited contact between clusters on aligned fibers suggests that topography overrides cellular chemoattractant cues (as proposed by (Sheets et al., 2013), though communication may still occur (Nahum et al., 2023). The 60-fold increase in connexin 43 (Cx43) mRNA expression on random fibers indicated robust cell–cell signaling, consistent with previous findings linking gap junctions to substrate stiffness (Zhou et al., 2020) and possibly to regulation of proliferation (Genet et al., 2023). It could also be possibly associated with regulation of proliferation or specific SH-SY5Y characteristics, previously shown to maintain their phenotype when seeded in high density, irrespective of 2D or 3D growth conditions (Consales et al., 2021). This dramatic upregulation could indicate intensified cell–cell communication, likely as a compensatory mechanism to alleviate mechanical and topographical stress. This may reflect a cellular strategy to maintain synchrony and paracrine signaling under less organized, more spatially dynamic conditions.

Notably, the thickness of the pseudospheroids is a limiting factor for immunofluorescence visualization. Therefore, to visualize the whole spheroid required extended permeabilization (2 h post-fixation). Aligned fibers retained attached pseudospheroids post-treatment, while in some cases random fibers partially failed, suggesting stronger cell-material interactions. This finding is further supported by elevated DCX expression on aligned fibers, consistent with reports linking DCX to neural migration (Ayanlaja et al., 2017; Gleeson et al., 1999; Daou et al., 2005) Beta actin mRNA increased on random fibers, suggestive of increased cell migration, as proposed in another study using MSCs (Werner et al., 2017), linking actin dynamics to surface curvature. FN1 reduction at both mRNA and protein levels further supports increased migration (Tan et al., 2021). Acetylated tubulin expression was comparable between fiber types, aligning with findings associating it with neurite extension (Cappelletti et al., 2021). Therefore, markers of immature neuronal states and migration, were significantly upregulated on aligned fibers, suggesting these topographies may better support directed neurogenesis and structural maturation. In contrast, higher β-actin and *FN1* expression on random fibers suggested increased cell remodeling activity.

Nuclear morphology followed fiber orientation, consistent with prior work (Lele et al., 2018; Huang et al., 2016; Doolin et al., 2019). Nuclei followed the surface directional cues irrespective of cell seeding density when in contact with the surface as previously shown by others (Huynh and Holsinger, 2023). Notably, more apoptotic nuclei appeared on aligned fibers, correlating with decreased DHCR24 expression, a protective enzyme against apoptosis (Lu et al., 2023). LDH assays, however, did not indicate elevated cytotoxicity, reinforcing the observation that apoptosis was likely due to over-confluence inducing mechanical or differentiation stress.

FGF2, a proliferative and anti-apoptotic cytokine, remained stable on fibers but doubled on glass (Sun et al., 2018; Wu et al., 2024; Lavenius et al., 1994), suggesting higher proliferation and less differentiation on glass. These results are supported by earlier studies showing increased differentiation on fibrous scaffolds (Wu et al., 2018; Boku et al., 2013; Scheil et al., 1994). Additionally, gradients of acidic FGF (FGF1), another member of the FGF family, have been shown to promote neurite extension both *in vitro* and *in vivo* (Jin et al., 2023), further supporting the role of FGF signaling in scaffold-guided neuroregeneration. In this study, detection of FGF2 but not FGF1 is indicative of the neurotrophic and anti-apoptotic role of FGF signaling, especially on glass surfaces where FGF2 increased significantly.

Adiponectin (ADPN) mRNA expression remained stable but showed lower fold changes on fibers compared to glass, suggesting minimal metabolic stress under fibrous conditions (Bloemer et al., 2018; Abgrall et al., 2022). LDH data confirmed cell viability within pseudospheroids, ruling out hypoxia-induced core death commonly observed in spheroid cultures (Xiang et al., 2000; Zhao et al., 2022).

Prior studies have demonstrated the benefits of incorporating fiber scaffolds into spheroids, improving ECM deposition and neural differentiation (Ahmad et al., 2017; Ahmad et al., 2020; Sharma et al., 2025). More cell differentiation was previously documented, when fibrous material was incorporated to spheroid formation, assessed by ECM, connectivity and differentiation markers, such as FN1 (Paten et al., 2019), Cx43 and SOX2 (Ahmad et al., 2017; Ahmad et al., 2020). Similarly, another *in vitro* study has shown Liu et al. (2023) reported enhanced sciatic nerve regeneration using randomly organized PCL fibers (Liu et al., 2023). The work presented here confirms increased expression of β3-tubulin and actin on random fibers, and of nestin and doublecortin on aligned fibers, indicating distinct differentiation pathways influenced by fiber topography.

Among others, alignment of scaffolding supports cell elongation and neurite-like protrusions, providing topographical cues to promote adhesion and maturation. In the neuroregeneration field, particularly in the context of spinal cord and peripheral nerve repair, fiber alignment has been studied since the mid-2000s as a strategy to facilitate healing in these structurally complex tissues (Yim et al., 2005; Schnell et al., 2007; Chew et al., 2008; Woods et al., 2022). The neurite-like extensions and their deeper integration transversing the aligned scaffolds observed in our study are consistent with mechanisms of neuronal maturation. Previous studies have demonstrated that aligned fibers promote neurite outgrowth (Yao et al., 2009; Ghollasi and Poormoghadam, 2022; Elashnikov et al., 2019; Yadav and Majumder, 2022; Yao et al., 2023). Our findings showed increased expression of the immature neuronal and migration markers DCX (Lendahl et al., 1990) and nestin on aligned fibers. Along with the enhanced presence of neurite-like protrusions, this suggests a shift toward a more neuron-like phenotype on aligned fibers and glass, while cells on random fibers retained a more stem-like character (Liu et al., 2018; Forster et al., 2016). However, the decrease in nestin (NES) expression by day 7 (D7) indicates progression toward maturation. The overall decline in neural cell adhesion molecule (NCAM) expression across all substrates likely reflects progression toward neuronal maturation. However, its more stable expression on aligned fibers suggests that alignment may help sustain a transitional progenitor state, consistent with observations in Schwann cells cultured on PCL (Chew et al., 2008).

Cx31, linked to neurite extension (Unsworth et al., 2007), was upregulated on aligned fibers and glass. Cx43 and fibronectin (FN1) expression have also been linked through focal adhesion kinase signaling (Zhou et al., 2020). In our study, FN1 expression decreased only on random fibers, potentially limiting collagen III formation. Collagen I was undetectable on glass and weakly expressed on both fibrous surfaces. Collagen III was present in all samples, contrary to earlier findings (Scarpa et al., 1987) and supporting more recent evidence that fiber orientation influences collagen deposition (Lee et al., 2005; Chen et al., 2024). Expression of β3-tubulin and Cx43 proteins decreased on aligned fibers and showed only minimal increases on glass, following similar trends in mRNA expression. This pattern supports a potential regulatory relationship between β3-tubulin and Cx43, as previously described (Giepmans et al., 2001). Aligned collagen-rich ECMs are associated with invasive behaviors in tumors (Ray and Provenzano, 2021). SOX2 localization was nuclear on aligned fibers and glass, indicating maintained stemness (Van Schaijik et al., 2018; Ferlemann et al., 2017), while its cytoplasmic presence on random fibers could imply secretion in the form of exosomes (Vaidya and Sugaya, 2020).

Although the findings provide new insights to the field, the study has limitations. First, we used polycaprolactone (PCL), which is hydrophobic and degrades slowly. We chose PCL for its electrospinnability, stability, and reproducibility in generating controlled fiber morphologies, that enabled us to isolate the effects of topography on SH-SY5Y cell behaviour. While this ensured experimental control and comparability with prior studies, it limits direct translational relevance. Nevertheless, the insights gained regarding fiber alignment and morphology are likely transferable to other biodegradable polymers, such as PLGA or natural blends with faster degradation rates. Second, subtle differences in fiber diameter between the aligned and random scaffolds were not further titrated. These dimensional variations may have acted as confounding factors and could have contributed to the observed cellular responses. Furthermore, we did not examine exosomal release or perform mechanical testing of cell–scaffold interactions. Previous work shows that PCL elasticity is direction-dependent, with increased stiffness when pulled parallel to fiber alignment (Delaine-Smith et al., 2021; Doustgani et al., 2011). Future studies should investigate how these properties influence scaffold–cell integration. Moreover, while we observed stronger cluster retention on aligned fibers following the prolonged Triton X-100 permeabilization, we did not quantitatively measure cell–scaffold adhesion strength. This qualitative observation merits further investigation, especially in the context of incorporating decellularized matrices into bioengineered scaffolds to enhance anchorage and remodeling potential.

Another limitation is that this study did not assess critical aspects of neuronal maturation, such as late neuronal differentiation or axonal outgrowth. While we addressed early neuronal and adhesion-related markers (DCX, βIII-tubulin, and NCAM) and found their expression modulated by scaffold topography, our data do not directly capture axonal extension. Also, we did not perform migration assays or other functional experiments to contextualize the expression of nestin and doublecortin. While we interpret them as markers of early neuronal lineage and cytoskeletal remodeling, they may also be influenced by stress or dedifferentiation. Similarly, the marked upregulation of connexin 43 observed on random fibers could reflect hypoxic signaling or stress responses in dense clusters, in addition to our proposed compensatory signaling mechanism. We employed SH-SY5Y cells, a neuroblastoma-derived line widely used in neural research. Although their use is practical and well characterized, they do not fully recapitulate primary or stem-cell-derived neurons and may limit the generalizability of our findings. Future studies can address this, employing human embryonic stem cell–or IPSC- based neuronal differentiation models on the scaffolds. Additionally, the application of advanced methods, such as spatial transcriptomics, could systematically map how fiber alignment and morphology influence differentiation trajectories and neuronal architecture.

In this study, we intentionally employed a high initial seeding density to mimic later stages of wound healing, aiming to facilitate extensive cell–cell interactions and paracrine signaling. While this setup may have masked subtle topography-dependent effects on initial attachment or very early differentiation, it enabled the robust formation of pseudospheroids.

Hereby, we report the characterization of neural, scaffold-anchored, high-density multicellular pseudospheroids, distinct from conventional suspension spheroids. This also highlights a unique tissue-engineering paradigm; unlike suspension spheroids, which lack a structural anchor, scaffold-attached aggregates can integrate topographical and material-derived cues to promote tissue-like organization. Our findings further show that high-density seeding induced rapid pseudospheroid formation and distinct differentiation trajectories depending on fiber alignment. Random fibers supported efficient surface coverage, migration, and intercellular communication, whereas aligned fibers promoted deeper anchorage, expression of neuronal lineage markers, and more mature-like phenotypes. These results suggest that fiber alignment not only directs cell organization but may also influence the balance between early cell network behaviour and progression toward neuronal maturation.

A promising future strategy may lie in the design of hierarchical or composite scaffolds that integrate both random and aligned fiber domains. Such hybrid architectures could combine the rapid surface colonization and communication enabled by random fibers with the axonal alignment, anchorage, and maturation promoted by aligned fibers. These hybrid approaches could be adapted to neural tissue repair systems and may provide a powerful platform to coordinate scaffold coverage with functional differentiation, ultimately advancing scaffold-based strategies for neural tissue reconstruction. Further research should investigate how material-derived cues and dynamic spatiotemporal signaling coordinate the reconstruction and functional restoration of neural tissues and networks following injury.

## Data Availability

The raw data supporting the conclusions of this article will be made available by the authors, without undue reservation.

## References

[B1] Abgrall A. Poizat G. Prevost M. Riffault L. De La Barrera L. Hanine R. (2022). Evidence for the neuronal expression and secretion of adiponectin. Cells 11, 2725. 10.3390/cells11172725 36078135 PMC9454681

[B2] Ahmad T. Lee J. Shin Y. M. Shin H. J. Madhurakat Perikamana S. K. Park S. H. (2017). Hybrid-spheroids incorporating ECM like engineered fragmented fibers potentiate stem cell function by improved cell/cell and cell/ECM interactions. Acta biomater. 64, 161–175. 10.1016/j.actbio.2017.10.022 29037892

[B3] Ahmad T. Byun H. Lee J. Madhurakat Perikamana S. K. Shin Y. M. Kim E. M. (2020). Stem cell spheroids incorporating fibers coated with adenosine and polydopamine as a modular building blocks for bone tissue engineering. Biomaterials 230, 119652. 10.1016/j.biomaterials.2019.119652 31787333

[B4] Ayanlaja A. A. Xiong Y. Gao Y. Ji G. Tang C. Abdullah Z. (2017). Distinct features of doublecortin as a marker of neuronal migration and its implications in cancer cell mobility. Front. Mol. Neurosci. 10, 199–270229. 10.3389/fnmol.2017.00199 28701917 PMC5487455

[B5] Bacakova L. Zikmundova M. Pajorova J. Broz A. Filova E. Blanquer A. (2019). Nanofibrous scaffolds for skin tissue engineering and wound healing based on synthetic polymers. In: Applications of nanobiotechnology. London, UK: IntechOpen.

[B6] Blahnová V. Vocetková K. Hlinkova J. Divin R. Amler E. Filová E. (2020). PCL scaffold for osteochondral defect treatment. Key Eng. Mater. 834, 141–147. 10.4028/www.scientific.net/kem.834.141

[B7] Bloemer J. Pinky P. D. Govindarajulu M. Hong H. Judd R. Amin R. H. (2018). Role of adiponectin in central nervous system disorders. Neural Plast. 2018, 1–15. 10.1155/2018/4593530 30150999 PMC6087588

[B8] Boku S. Nakagawa S. Toda H. Kato A. Takamura N. Omiya Y. (2013). ROCK2 regulates bFGF-induced proliferation of SH-SY5Y cells through GSK-3β and β-catenin pathway. Brain Res. 1492, 7–17. 10.1016/j.brainres.2012.11.034 23211630

[B9] Braťka P. Fenclová T. Hlinková J. Uherková L. Šebová E. Hefka Blahnová V. (2022). The preparation and biological testing of novel wound dressings with an encapsulated antibacterial and antioxidant substance. Nanomaterials 12, 3824. 10.3390/nano12213824 36364600 PMC9656126

[B10] Brown B. N. Badylak S. F. (2014). Extracellular matrix as an inductive scaffold for functional tissue reconstruction. Transl. Res. J. laboratory Clin. Med. 163, 268–285. 10.1016/j.trsl.2013.11.003 24291155 PMC4203714

[B115] Cai J. H. Deng S. Kumpf S. W. Lee P. A. Zagouras P. Ryan R. (2007). Validation of rat reference genes for improved quantitative gene expression analysis using low density arrays. BioTechniques 42 (4), 503–511. 10.2144/000112400 17489238

[B11] Çapkin M. Çakmak S. Kurt F. Ö. Gumusderelioglu M. Şen B. H. Türk B. T. (2012). Random/Aligned electrospun PCL/PCL-collagen nanofibrous membranes: comparison of neural differentiation of rat AdMSCs and BMSCs. Biomed. Mater. 7, 045013. 10.1088/1748-6041/7/4/045013 22652636

[B12] Cappelletti G. Calogero A. M. Rolando C. (2021). Microtubule acetylation: a reading key to neural physiology and degeneration. Neurosci. Lett. 755, 135900. 10.1016/j.neulet.2021.135900 33878428

[B13] Carletti E. Motta A. Migliaresi C. (2011). Scaffolds for tissue engineering and 3D cell culture. Methods Mol. Biol. 695, 17–39. 10.1007/978-1-60761-984-0_2 21042963

[B14] Chamanehpour E. Thouti S. Rubahn H.-G. Dolatshahi-Pirouz A. Mishra Y. K. (2024). Smart nanofibers: synthesis, properties, and scopes in future advanced technologies. Adv. Mater. Technol. 9, 2301392. 10.1002/admt.202301392

[B15] Chan B. P. Leong K. W. (2008). Scaffolding in tissue engineering: general approaches and tissue-specific considerations. Eur. Spine J. 17, 467–479. 10.1007/s00586-008-0745-3 19005702 PMC2587658

[B16] Chen Y. Dong X. Shafiq M. Myles G. Radacsi N. Mo X. (2022). Recent advancements on three-dimensional electrospun nanofiber scaffolds for tissue engineering. Adv. Fiber Mater. 4, 959–986. 10.1007/s42765-022-00170-7

[B17] Chen Y. Lu W. Zhou Y. Hu Z. Wu H. Gao Q. (2024). A spatiotemporal controllable biomimetic skin for accelerating wound repair. Small 20, 2310556. 10.1002/smll.202310556 38386291

[B18] Chew S. Y. Mi R. Hoke A. Leong K. W. (2008). The effect of the alignment of electrospun fibrous scaffolds on schwann cell maturation. Biomaterials 29, 653–661. 10.1016/j.biomaterials.2007.10.025 17983651 PMC2713097

[B19] Christopherson G. T. Song H. Mao H. Q. (2009). The influence of fiber diameter of electrospun substrates on neural stem cell differentiation and proliferation. Biomaterials 30, 556–564. 10.1016/j.biomaterials.2008.10.004 18977025

[B20] Consales C. Butera A. Merla C. Pasquali E. Lopresto V. Pinto R. (2021). Exposure of the SH-SY5Y human neuroblastoma cells to 50-Hz magnetic field: Comparison between two-dimensional (2D) and three-dimensional (3D) *in vitro* cultures. Mol. Neurobiol. 58, 1634–1649. 10.1007/s12035-020-02192-x 33230715 PMC7932966

[B21] Daou M. C. Smith T. W. Litofsky N. S. Hsieh C. C. Ross A. H. (2005). Doublecortin is preferentially expressed in invasive human brain tumors. Acta Neuropathol. 110, 472–480. 10.1007/s00401-005-1070-0 16195916

[B22] Dash T. K. Konkimalla V. B. (2012). Poly-є-caprolactone based formulations for drug delivery and tissue engineering: a review. J. Control. 158, 15–33. 10.1016/j.jconrel.2011.09.064 21963774

[B23] Delaine-Smith R. M. Hann A. J. Green N. H. Reilly G. C. (2021). Electrospun fiber alignment guides osteogenesis and matrix organization differentially in two different osteogenic cell types. Front. Bioeng. Biotechnol. 9, 672959. 10.3389/fbioe.2021.672959 34760876 PMC8573409

[B24] Doolin M. T. Ornstein T. S. Stroka K. M. (2019). Nuclear deformation in response to mechanical confinement is cell type dependent. Cells 8, 427. 10.3390/cells8050427 31072066 PMC6563141

[B25] Doss B. L. Pan M. Gupta M. Grenci G. Mège R. M. Lim C. T. (2020). Cell response to substrate rigidity is regulated by active and passive cytoskeletal stress. Proc. Natl. Acad. Sci. U. S. A. 117, 12817–12825. 10.1073/pnas.1917555117 32444491 PMC7293595

[B26] Doustgani A. Vasheghani-Farahani E. Soleimani M. Hashemi-Najafabadi S. (2011). Preparation and characterization of aligned and random nanofibrous nanocomposite scaffolds of poly (vinyl alcohol), poly (e-Caprolactone) and nanohydroxyapatite. Int. J. Nanosci. Nanotechnol. 7, 127–132. Available online at: https://www.ijnnonline.net/article_3936_a8878ff5982c9f66b5d8cf52694a46af.pdf.

[B27] Duan X. Chen H. L. Guo C. (2022). Polymeric nanofibers for drug delivery applications: a recent review. J. Mater. Sci. Mater. Med. 33, 78. 10.1007/s10856-022-06700-4 36462118 PMC9719450

[B28] Dziemidowicz K. Kellaway S. C. Guillemot-Legris O. Matar O. Trindade R. P. Roberton V. H. (2023). Development of ibuprofen-loaded electrospun materials suitable for surgical implantation in peripheral nerve injury. Biomater. Adv. 154, 213623. 10.1016/j.bioadv.2023.213623 37837905

[B29] D’Amato A. R. Puhl D. L. Ziemba A. M. Johnson C. D. L. Doedee J. Bao J. (2019). Exploring the effects of electrospun fiber surface nanotopography on neurite outgrowth and branching in neuron cultures. PLoS One 14, e0211731. 10.1371/journal.pone.0211731 30716106 PMC6361509

[B30] Edgar L. Mcnamara K. Wong T. Tamburrini R. Katari R. Orlando G. (2016). Heterogeneity of scaffold biomaterials in tissue engineering. Mater. 9, 332. 10.3390/ma9050332 28773457 PMC5503070

[B31] Elashnikov R. Rimpelová S. Děkanovský L. Švorčík V. Lyutakov O. (2019). Polypyrrole-coated cellulose nanofibers: influence of orientation, coverage and electrical stimulation on SH-SY5Y behavior. J. Mater. Chem. B 7, 6500–6507. 10.1039/c9tb01300h 31576390

[B32] Eleftheriadou D. Kesidou D. Moura F. Felli E. Song W. (2020). Redox-responsive nanobiomaterials-based therapeutics for neurodegenerative diseases. Small 16, 1907308. 10.1002/smll.201907308 32940007

[B33] Fahimirad S. Fahimirad Z. Sillanpää M. (2021). Efficient removal of water bacteria and viruses using electrospun nanofibers. Sci. Total Environ. 751, 141673. 10.1016/j.scitotenv.2020.141673 32866832 PMC7428676

[B34] Ferlemann F. C. Menon V. Condurat A. L. RößLER J. Pruszak J. (2017). Surface marker profiling of SH-SY5Y cells enables small molecule screens identifying BMP4 as a modulator of neuroblastoma differentiation. Sci. Rep. 7, 13612–13614. 10.1038/s41598-017-13497-8 29051534 PMC5648761

[B35] Ferraris S. Spriano S. Scalia A. C. Cochis A. Rimondini L. Cruz-Maya I. (2020). Topographical and biomechanical guidance of electrospun fibers for biomedical applications. Polymers 12 (12), 2896. 10.3390/polym12122896 33287236 PMC7761715

[B36] Fiore N. J. Tamer-Mahoney J. D. Beheshti A. Nieland T. J. F. Kaplan D. L. (2022). 3D biocomposite culture enhances differentiation of dopamine-like neurons from SH-SY5Y cells: a model for studying parkinson's disease phenotypes. Biomaterials 290, 121858. 10.1016/j.biomaterials.2022.121858 36272218

[B37] Forster J. I. Köglsberger S. Trefois C. Boyd O. Baumuratov A. S. Buck L. (2016). Characterization of differentiated SH-SY5Y as neuronal screening model reveals increased oxidative vulnerability. J. Biomol. Screen. 21, 496–509. 10.1177/1087057115625190 26738520 PMC4904349

[B38] Genet N. Genet G. Chavkin N. W. Paila U. Fang J. S. Vasavada H. H. (2023). Connexin 43-mediated neurovascular interactions regulate neurogenesis in the adult brain subventricular zone. Cell Rep. 42, 112371. 10.1016/j.celrep.2023.112371 37043357 PMC10564973

[B39] Gerardo-Nava J. Führmann T. Klinkhammer K. Seiler N. Mey J. Klee D. (2009). Human neural cell interactions with orientated electrospun nanofibers *in vitro* . Nanomedicine (Lond) Engl. 4, 11–30. 10.2217/17435889.4.1.11 19093893

[B40] Ghollasi M. Poormoghadam D. (2022). Enhanced neural differentiation of human-induced pluripotent stem cells on aligned laminin-functionalized polyethersulfone nanofibers; a comparison between aligned and random fibers on neurogenesis. J. Biomed. Mater. Res. Part A 110, 672–683. 10.1002/jbm.a.37320 34651431

[B41] Giepmans B. N. G. Verlaan I. Hengeveld T. Janssen H. Calafat J. Falk M. M. (2001). Gap junction protein connexin-43 interacts directly with microtubules. Curr. Biol. CB 11, 1364–1368. 10.1016/s0960-9822(01)00424-9 11553331

[B42] Gleeson J. G. Lin P. T. Flanagan L. A. Walsh C. A. (1999). Doublecortin is a microtubule-associated protein and is expressed widely by migrating neurons. Neuron 23, 257–271. 10.1016/s0896-6273(00)80778-3 10399933

[B43] Gupta D. Venugopal J. Prabhakaran M. P. Dev V. R. G. Low S. Choon A. T. (2009). Aligned and random nanofibrous substrate for the *in vitro* culture of schwann cells for neural tissue engineering. Acta biomater. 5, 2560–2569. 10.1016/j.actbio.2009.01.039 19269270

[B44] Haidar M. Eroglu H. (2017). Nanofibers: new insights for drug delivery and tissue engineering. Curr. Top. Med. Chem. 17, 1564–1579. 10.2174/1568026616666161222102641 28017155

[B45] Hall M. S. Alisafaei F. Ban E. Feng X. Hui C. Y. Shenoy V. B. (2016). Fibrous nonlinear elasticity enables positive mechanical feedback between cells and ECMs. Proc. Natl. Acad. Sci. U. S. A. 113, 14043–14048. 10.1073/pnas.1613058113 27872289 PMC5150395

[B46] Hammarback J. A. Letourneau P. C. (1986). Neurite extension across regions of low cell-substratum adhesivity: implications for the guidepost hypothesis of axonal pathfinding. Dev. Biol. 117, 655–662. 10.1016/0012-1606(86)90334-9 3758485

[B47] Hernández-Parra H. Cortés H. Romero-Montero A. Borbolla-Jiménez F. V. Magaña J. J. Del Prado-Audelo M. L. (2024). Polymeric nanoparticles decorated with fragmented chitosan as modulation systems for neuronal drug uptake. Carbohydr. Polym. 336, 122121. 10.1016/j.carbpol.2024.122121 38670753

[B48] Horwacik I. (2022). The extracellular matrix and neuroblastoma cell communication—A complex interplay and its therapeutic implications. Cells 11 (11), 3172. 10.3390/cells11193172 36231134 PMC9564247

[B49] Huang C. Y. Hu K. H. Wei Z. H. (2016). Comparison of cell behavior on pva/pva-gelatin electrospun nanofibers with random and aligned configuration. Sci. Rep. 6, 37960. 10.1038/srep37960 27917883 PMC5137148

[B50] Huynh Q. S. Holsinger R. M. D. (2023). Fiber and electrical field alignment increases BDNF expression in SH-SY5Y cells following electrical stimulation. Pharmaceuticals 16, 138. 10.3390/ph16020138 37259290 PMC9960882

[B51] Ibrahim H. M. Klingner A. (2020). A review on electrospun polymeric nanofibers: production parameters and potential applications. Polym. Test. 90, 106647. 10.1016/j.polymertesting.2020.106647

[B52] Jin B. Yu Y. Lou C. Zhang X. Gong B. Chen J. (2023). Combining a density gradient of biomacromolecular nanoparticles with biological effectors in an electrospun fiber-based nerve guidance conduit to promote peripheral nerve repair. Adv. Sci. 10, 2203296. 10.1002/advs.202203296 36494181 PMC9896046

[B53] Jin M. Xie M. Liu Y. Song H. Zhang M. Li W. (2024). Circulating miR-30e-3p induces disruption of neurite development in SH-SY5Y cells by targeting ABI1, a novel biomarker for schizophrenia. J. Psychiatric Res. 174, 84–93. 10.1016/j.jpsychires.2024.04.005 38626565

[B54] Jung T. W. Lee J. Y. Shim W. S. Kang E. S. Kim J. S. Ahn C. W. (2006). Adiponectin protects human neuroblastoma SH-SY5Y cells against acetaldehyde-induced cytotoxicity. Biochem. Pharmacol. 72, 616–623. 10.1016/j.bcp.2006.05.013 16814256

[B55] Katti D. S. Robinson K. W. Ko F. K. Laurencin C. T. (2004). Bioresorbable nanofiber-based systems for wound healing and drug delivery: optimization of fabrication parameters. J. Biomed. Mater. Res 70, 286–296. 10.1002/jbm.b.30041 15264311

[B56] Kellaway S. C. Ullrich M. M. Dziemidowicz K. (2024). Electrospun drug-loaded scaffolds for nervous system repair. WIREs Nanomedicine Nanobiotechnol 16, e1965. 10.1002/wnan.1965 38740385

[B57] Khil M. S. Cha D. I. Kim H. Y. Kim I. S. Bhattarai N. (2003). Electrospun nanofibrous polyurethane membrane as wound dressing. J. Biomed. Mater. Res 67, 675–679. 10.1002/jbm.b.10058 14598393

[B58] Kim J. I. Kim C. S. Park C. H. (2018). Harnessing nanotopography of electrospun nanofibrous nerve guide conduits (NGCs) for neural tissue engineering. Adv. Exp. Med. Biol. 1078, 395–408. 10.1007/978-981-13-0950-2_20 30357634

[B59] Lavenius E. Parrow V. Nånberg E. Påhlman S. (1994). Basic FGF and IGF-I promote differentiation of human SH-SY5Y neuroblastoma cells in culture. Growth factors. 10, 29–39. 10.3109/08977199409019601 7514011

[B60] Lee C. H. Shin H. J. Cho I. H. Kang Y.-M. Kim I. A. Park K.-D. (2005). Nanofiber alignment and direction of mechanical strain affect the ECM production of human ACL fibroblast. Biomaterials 26, 1261–1270. 10.1016/j.biomaterials.2004.04.037 15475056

[B61] Lele T. P. Dickinson R. B. Gundersen G. G. (2018). Mechanical principles of nuclear shaping and positioning. J. Cell Biol. 217, 3330–3342. 10.1083/jcb.201804052 30194270 PMC6168261

[B62] Lendahl U. Zimmerman L. B. Mckay R. D. G. (1990). CNS stem cells express a new class of intermediate filament protein. Cell 60, 585–595. 10.1016/0092-8674(90)90662-x 1689217

[B63] Linkert M. Rueden C. T. Allan C. Burel J. M. Moore W. Patterson A. (2010). Metadata matters: access to image data in the real world. J. Cell Biol. 189, 777–782. 10.1083/jcb.201004104 20513764 PMC2878938

[B64] Liu J. Y. W. Matarin M. Reeves C. Mcevoy A. W. Miserocchi A. Thompson P. (2018). Doublecortin-expressing cell types in temporal lobe epilepsy. Acta neuropathol. Commun. 6, 60. 10.1186/s40478-018-0566-5 30005693 PMC6045867

[B65] Liu C. Liu D. Zhang X. Hui L. Zhao L. (2023). Nanofibrous polycaprolactone/amniotic membrane facilitates peripheral nerve regeneration by promoting macrophage polarization and regulating inflammatory microenvironment. Int. Immunopharmacol. 121, 110507. 10.1016/j.intimp.2023.110507 37356125

[B66] Lu Z. Wang H. Zhang X. Huang X. Jiang S. Li Y. (2023). High fat diet induces brain injury and neuronal apoptosis via down-regulating 3-β hydroxycholesterol 24 reductase (DHCR24). Cell tissue Res. 393, 471–487. 10.1007/s00441-023-03804-3 37458798

[B67] Marques-Almeida T. Lanceros-Mendez S. Ribeiro C. (2024). State of the art and current challenges on electroactive biomaterials and strategies for neural tissue regeneration. Adv. Healthc. Mater. 13, 2301494. 10.1002/adhm.202301494 37843074

[B68] Mcmurtrey R. J. (2014). Patterned and functionalized nanofiber scaffolds in three-dimensional hydrogel constructs enhance neurite outgrowth and directional control. J. neural Eng. 11, 066009. 10.1088/1741-2560/11/6/066009 25358624

[B69] Melaiye A. Sun Z. Hindi K. Milsted A. Ely D. Reneker D. H. (2005). Silver(I)-imidazole cyclophane gem-diol complexes encapsulated by electrospun tecophilic nanofibers: formation of nanosilver particles and antimicrobial activity. J. Am. Chem. Soc. 127, 2285–2291. 10.1021/ja040226s 15713108

[B70] Mungenast L. Nieminen R. Gaiser C. Faia-Torres A. B. Rühe J. Suter-Dick L. (2023). Electrospun decellularized extracellular matrix scaffolds promote the regeneration of injured neurons. Biomaterials Biosyst. 11, 100081. 10.1016/j.bbiosy.2023.100081 37427248 PMC10329103

[B71] Nahum A. Koren Y. Ergaz B. Natan S. Miller G. Tamir Y. (2023). Inference of long-range cell-cell force transmission from ECM remodeling fluctuations. Commun. Biol. 6, 811–818. 10.1038/s42003-023-05179-1 37537232 PMC10400639

[B114] Nekooki-Machida Y. Hagiwara H. (2020). Role of tubulin acetylation in cellular functions and diseases. Med. Mol. Morphol. 53 (4), 191–197. 10.1007/s00795-020-00260-8 32632910

[B72] Papadopoulou E. L. Samara A. Barberoglou M. Manousaki A. Pagakis S. N. Anastasiadou E. (2010). Silicon scaffolds promoting three-dimensional neuronal web of cytoplasmic processes. Tissue Eng. - Part C. Methods 16, 497–502. 10.1089/ten.tec.2009.0216 19686056

[B73] Park D. Lee S. J. Choi D. K. Park J. W. (2023). Therapeutic agent-loaded fibrous scaffolds for biomedical applications. Pharmaceutics 15, 1522. 10.3390/pharmaceutics15051522 37242764 PMC10223832

[B74] Paten J. A. Martin C. L. Wanis J. T. Siadat S. M. Figueroa-Navedo A. M. Ruberti J. W. (2019). Molecular Interactions between Collagen and Fibronectin: a Reciprocal Relationship that Regulates *de novo* Fibrillogenesis. Chem 5, 2126–2145. 10.1016/j.chempr.2019.05.011

[B75] Randhawa A. Dutta S. D. Ganguly K. Patil T. V. Lim K. T. (2024). Manufacturing 3D biomimetic tissue: a strategy involving the integration of electrospun nanofibers with a 3D-Printed framework for enhanced tissue regeneration. Small 20, 2309269. 10.1002/smll.202309269 38308170

[B76] Ray A. Provenzano P. P. (2021). Aligned forces: origins and mechanisms of cancer dissemination guided by ECM architecture. Curr. Opin. Cell Biol. 72, 63. 10.1016/j.ceb.2021.05.004 34186415 PMC8530881

[B77] Saghir S. Hlinkova J. Samara A. Schiavone G. (2025). Protocol for fabricating a reusable plate-well insert with a 3D-printed mounter and hydrogel scaffolds for 3D cell culture and functional assays. Star. Protoc. 6, 104014. 10.1016/j.xpro.2025.104014 40782348 PMC12356002

[B78] Samara A. Galbiati M. Luciani P. Deledda C. Messi E. Peri A. (2014). Altered expression of 3-betahydroxysterol delta-24-reductase/selective alzheimer's disease indicator-1 gene in huntington's disease models. J. Endocrinol. investigation 37, 729–737. 10.1007/s40618-014-0098-1 24916565

[B79] Samara A. Spildrejorde M. Sharma A. Falck M. Leithaug M. Modafferi S. (2022). A multi-omics approach to visualize early neuronal differentiation from hESCs in 4D. iScience 25, 105279. 10.1016/j.isci.2022.105279 36304110 PMC9593815

[B80] Scarpa S. Modesti A. Triche T. J. (1987). Extracellular matrix synthesis by undifferentiated childhood tumor cell lines. Am. J. Pathology 129, 74–85. Available online at : https://pmc.ncbi.nlm.nih.gov/articles/instance/1899707/pdf/amjpathol00139-0081.pdf . 2821816 PMC1899707

[B81] Schaub N. J. D'Amato A. R. Mason A. Corr D. T. Harmon E. Y. Lennartz M. R. (2017). The effect of engineered nanotopography of electrospun microfibers on fiber rigidity and macrophage cytokine production. J Biomater Sci Polym Ed 28, 1303–1323. 10.1080/09205063.2017.1321345 28420296

[B82] Scheil S. Lüdecke G. Unsicker K. (1994). Interleukin-1 beta and interferon gamma interact with fibroblast growth factor-2 in the control of neuroblastoma cell proliferation and differentiation. Int. J. Dev. Neurosci. 12, 405–410. 10.1016/0736-5748(94)90024-8 7817783

[B83] Schiffman J. D. Schauer C. L. (2008). A review: electrospinning of biopolymer nanofibers and their applications. Polym. Rev. 48, 317–352. 10.1080/15583720802022182

[B84] Schindelin J. Arganda-Carreras I. Frise E. Kaynig V. Longair M. Pietzsch T. (2012). Fiji: an open-source platform for biological-image analysis. Nat. Methods 9, 676–682. 10.1038/nmeth.2019 22743772 PMC3855844

[B85] Schmid B. Schindelin J. Cardona A. Longair M. Heisenberg M. (2010). A high-level 3D visualization API for java and ImageJ. BMC Bioinforma. 11, 274–277. 10.1186/1471-2105-11-274 20492697 PMC2896381

[B86] Schnell E. Klinkhammer K. Balzer S. Brook G. Klee D. Dalton P. (2007). Guidance of glial cell migration and axonal growth on electrospun nanofibers of poly-ε-caprolactone and a collagen/poly-ε-caprolactone blend. Biomaterials 28, 3012–3025. 10.1016/j.biomaterials.2007.03.009 17408736

[B87] Senapati S. Tripathi K. Awad K. Rahimipour S. (2024). Multifunctional liposomes targeting Amyloid-β oligomers for early diagnosis and therapy of alzheimer's disease. Small 20, 2311670. 10.1002/smll.202311670 38461531

[B117] Sharma S. Agashe A. Hill J. C. Ganguly K. Sharma P. Richards T. D. (2025). Mechanical cues guide the formation and patterning of 3D spheroids in fibrous environments. PNAS Nexus 4 (9). 10.1093/pnasnexus/pgaf263 40978083 PMC12448454

[B89] Sheets K. Wang J. Meehan S. Sharma P. Ng C. Khan M. (2013). Cell-fiber interactions on aligned and suspended nanofiber scaffolds. J. Biomaterials Tissue Eng. 3, 355–368. 10.1166/jbt.2013.1105

[B90] Smith L. R. Cho S. Discher D. E. (2018). Stem cell differentiation is regulated by extracellular matrix mechanics. Physiology 33, 16–25. 10.1152/physiol.00026.2017 29212889 PMC5866410

[B91] Sousa M. G. C. Maximiano M. R. Costa R. A. Rezende T. M. B. Franco O. L. (2020). Nanofibers as drug-delivery systems for infection control in dentistry. Expert Opin. drug Deliv. 17, 919–930. 10.1080/17425247.2020.1762564 32401065

[B92] Sousa M. G. C. Rezende T. M. B. Franco O. L. (2021). Nanofibers as drug-delivery systems for antimicrobial peptides. Drug Discov. today 26, 2064–2074. 10.1016/j.drudis.2021.03.008 33741497

[B93] Spildrejorde M. Samara A. Sharma A. Leithaug M. Falck M. Modafferi S. (2023). Multi-omics approach reveals dysregulated genes during hESCs neuronal differentiation exposure to paracetamol. iScience 26, 107755. 10.1016/j.isci.2023.107755 37731623 PMC10507163

[B94] Suhito I. R. Angeline N. Lee K.-H. Kim H. Park C. G. Luo Z. (2021). A spheroid-forming hybrid gold nanostructure platform that electrochemically detects anticancer effects of curcumin in a multicellular brain cancer model. Small 17, 2002436. 10.1002/smll.202002436 32954643

[B95] Sun D. Wang W. Wang X. Wang Y. Xu X. Ping F. (2018). bFGF plays a neuroprotective role by suppressing excessive autophagy and apoptosis after transient global cerebral ischemia in rats. Cell death and Dis. 9, 172. 10.1038/s41419-017-0229-7 29416039 PMC5833346

[B96] Tan X. Gong W. Chen B. Gong B. Hua Z. Zhang S. (2021). Downregulation of fibronectin 1 attenuates ATRA-Induced inhibition of cell migration and invasion in neuroblastoma cells. Mol. Cell. Biochem. 476, 3601–3612. 10.1007/s11010-021-04113-5 34024029

[B97] Tian L. Prabhakaran M. P. Ramakrishna S. (2015). Strategies for regeneration of components of nervous system: scaffolds, cells and biomolecules. Regen. Biomater. 2, 31–45. 10.1093/rb/rbu017 26813399 PMC4669026

[B116] Unsworth H. C. Aasen T. McElwaine S. Kelsell D. P. (2007). Tissue-specific effects of wild-type and mutant connexin 31: a role in neurite outgrowth. Hum. Mol. Genet. 16 (2), 165–172. 10.1093/hmg/ddl452 17142249

[B98] Vaidya M. Sugaya K. (2020). Differential sequences and single nucleotide polymorphism of exosomal SOX2 DNA in cancer. PloS one 15, e0229309. 10.1371/journal.pone.0229309 32092088 PMC7039433

[B99] Van Schaijik B. Davis P. F. Wickremesekera A. C. Tan S. T. Itinteang T. (2018). Subcellular localisation of the stem cell markers OCT4, SOX2, NANOG, KLF4 and c-MYC in cancer: a review. J. Clin. Pathology 71, 88–91. 10.1136/jclinpath-2017-204815 29180509

[B100] Werner M. Blanquer S. B. G. Haimi S. P. Korus G. Dunlop J. W. C. Duda G. N. (2017). Surface curvature differentially regulates stem cell migration and differentiation via altered attachment morphology and nuclear deformation. Adv. Sci. 4, 1600347. 10.1002/advs.201600347 28251054 PMC5323878

[B101] Woodruff M. A. Hutmacher D. W. (2010). The return of a forgotten polymer—Polycaprolactone in the 21st century. Prog. Polym. Sci. 35, 1217–1256. 10.1016/j.progpolymsci.2010.04.002

[B102] Woods I. O'Connor C. Frugoli L. Kerr S. Gutierrez Gonzalez J. Stasiewicz M. (2022). Biomimetic scaffolds for spinal cord applications exhibit stiffness-dependent immunomodulatory and neurotrophic characteristics. Adv. Healthc. Mater. 11, 2101663. 10.1002/adhm.202101663 34784649

[B103] Wu Y. Wang Z. Cai P. Jiang T. Li Y. Yuan Y. (2018). Dual delivery of bFGF- and NGF-binding coacervate confers neuroprotection by promoting neuronal proliferation. Cell. physiology Biochem. 47, 948–956. 10.1159/000490139 29895019

[B104] Wu Y. Sun J. Lin Q. Wang D. Hai J. (2024). Sustained release of vascular endothelial growth factor A and basic fibroblast growth factor from nanofiber membranes reduces oxygen/glucose deprivation-induced injury to neurovascular units. Neural Regen. Res. 19, 887–894. 10.4103/1673-5374.382252 37843225 PMC10664103

[B105] Xiang Z. Hrabetova S. Moskowitz S. I. Casaccia-Bonnefil P. Young S. R. Nimmrich V. C. (2000). Long-term maintenance of mature hippocampal slices *in vitro* . J. Neurosci. Methods 98, 145–154. 10.1016/s0165-0270(00)00197-7 10880828

[B106] Yadav S. Majumder A. (2022). Biomimicked large-area anisotropic grooves from Dracaena sanderiana leaf enhances cellular alignment and subsequent differentiation. Bioinspiration. Biomimetics 17, 056002. 10.1088/1748-3190/ac7afe 35728757

[B107] Yang F. Murugan R. Wang S. Ramakrishna S. (2005). Electrospinning of nano/micro scale poly(L-lactic acid) aligned fibers and their potential in neural tissue engineering. Biomaterials 26, 2603–2610. 10.1016/j.biomaterials.2004.06.051 15585263

[B108] Yao L. O'Brien N. Windebank A. Pandit A. (2009). Orienting neurite growth in electrospun fibrous neural conduits. J. Biomed. Mater. Res 90, 483–491. 10.1002/jbm.b.31308 19130615

[B109] Yao C. Qiu Z. Li X. Zhu H. Li D. He J. (2023). Electrohydrodynamic printing of microfibrous architectures with cell-scale spacing for improved cellular migration and neurite outgrowth. Small 19, 2207331. 10.1002/smll.202207331 36775926

[B110] Yim E. K. F. Reano R. M. Pang S. W. Yee A. F. Chen C. S. Leong K. W. (2005). Nanopattern-induced changes in morphology and motility of smooth muscle cells. Biomaterials 26, 5405–5413. 10.1016/j.biomaterials.2005.01.058 15814139 PMC2376810

[B111] Zhang X. Meng Y. Gong B. Wang T. Lu Y. Zhang L. (2022). Electrospun nanofibers for manipulating soft tissue regeneration. J. Mater. Chem. 10, 7281–7308. 10.1039/d2tb00609j 35688128

[B112] Zhao Z. Chen X. Dowbaj A. M. Sljukic A. Bratlie K. Lin L. (2022). Organoids. Nat. Rev. Methods Prim. 2, 1–21. 10.1038/s43586-022-00174-y 37325195 PMC10270325

[B113] Zhou C. Zhang D. Du W. Zou J. Li X. Xie J. (2020). Substrate mechanics dictate cell-cell communication by gap junctions in stem cells from human apical papilla. Acta Biomater. 107, 178–193. 10.1016/j.actbio.2020.02.032 32105834

