# Supplementary figures and images for: Neural growth patterns: how random and aligned fibers guide 3D cell organization and pseudospheroid formation
Source: Front Bioeng Biotechnol. 2025 Oct 3;13:1659965. doi: 10.3389/fbioe.2025.1659965 (PMC12531216; doi:10.3389/fbioe.2025.1659965)

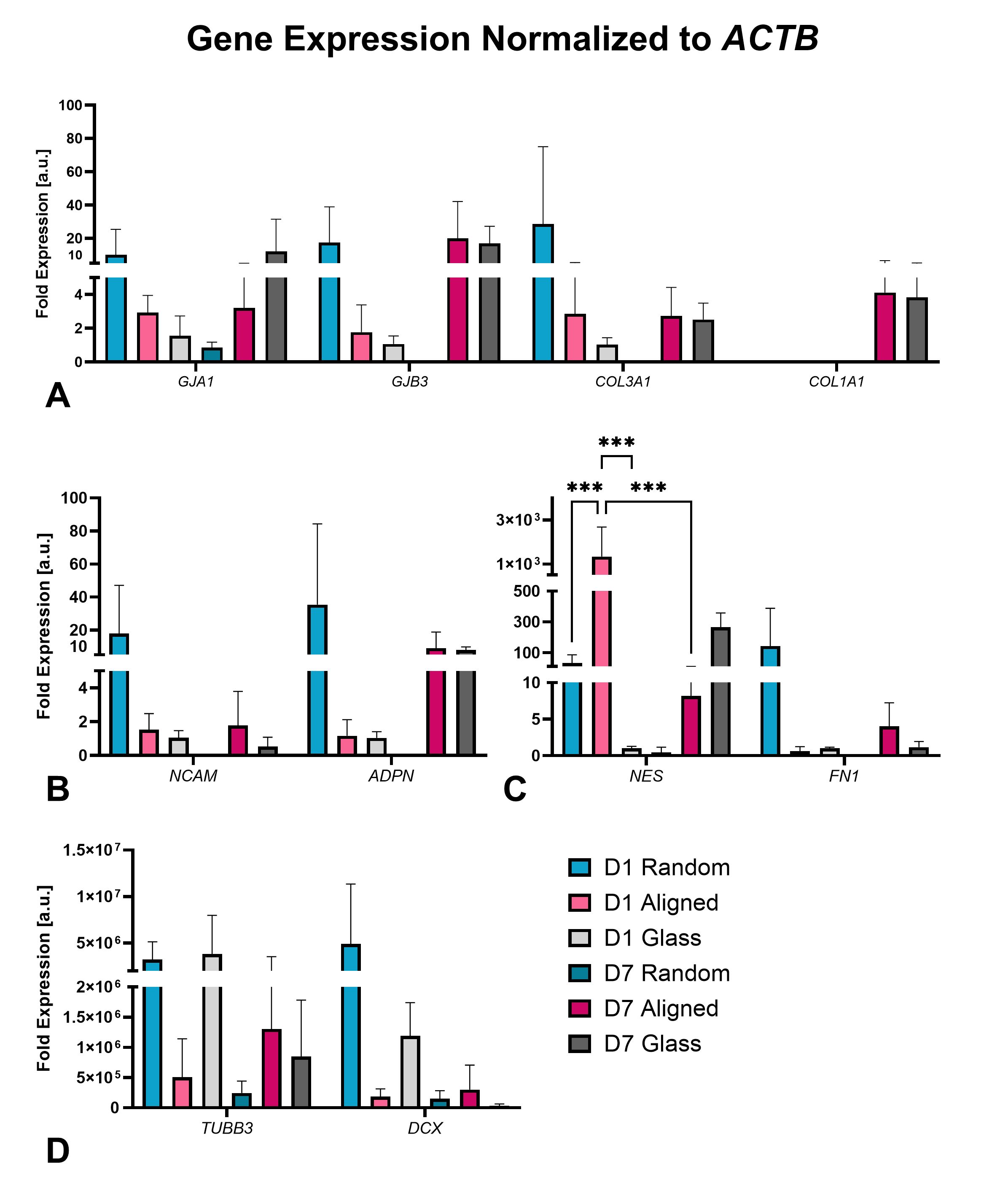

Supplement: Supplementary file 1 [file Image1.tif]
